# Privileged multi-target directed propargyl-tacrines combining cholinesterase and monoamine oxidase inhibition activities

**DOI:** 10.1080/14756366.2022.2122054

**Published:** 2022-09-21

**Authors:** Zofia Chrienova, Eugenie Nepovimova, Rudolf Andrys, Rafael Dolezal, Jana Janockova, Lubica Muckova, Lenka Fabova, Ondrej Soukup, Patrik Oleksak, Martin Valis, Jan Korabecny, José Marco-Contelles, Kamil Kuca

**Affiliations:** a Department of Chemistry, Faculty of Science, University of Hradec Kralove, Hradec Kralove, Czech Republic; b Biomedical Research Centre and Department of Neurology, University Hospital Hradec Kralove, Hradec Kralove, Czech Republic; c Department of Toxicology and Military Pharmacy, Faculty of Military Health Sciences, University of Defence, Hradec Kralove, Czech Republic; d Department of Pharmaceutical Chemistry and Pharmaceutical Analysis, Faculty of Pharmacy in Hradec Kralove, Charles University in Prague, Hradec Kralove, Czech Republic; e Faculty of Medicine in Hradec Kralove, Charles University in Prague, Hradec Kralove, Czech Republic; f Institute of General Organic Chemistry (CSIC), Laboratory of Medicinal Chemistry, Madrid, Spain

**Keywords:** Cholinesterase inhibitor, Alzheimer’s disease, monoamine oxidase inhibitor, propargyl amines, tacrine

## Abstract

Twenty-four novel compounds bearing tetrahydroacridine and *N*-propargyl moieties have been designed, synthesised, and evaluated *in vitro* for their anti-cholinesterase and anti-monoamine oxidase activities. Propargyltacrine **23** (IC_50_ = 21 nM) was the most potent acetylcholinesterase (AChE) inhibitor, compound **20** (IC_50_ = 78 nM) showed the best inhibitory human butyrylcholinesterase (*h*BChE) profile, and ligand **21** afforded equipotent and significant values on both ChEs (human AChE [*h*AChE]: IC_50_ = 0.095 ± 0.001 µM; *h*BChE: IC_50_ = 0.093 ± 0.003 µM). Regarding MAO inhibition, compounds **7**, **15**, and **25** demonstrated the highest inhibitory potential towards *h*MAO-B (IC_50_ = 163, 40, and 170 nM, respectively). In all, compounds **7**, **15**, **20**, **21**, **23**, and **25** exhibiting the most balanced pharmacological profile, were submitted to permeability and cell viability tests. As a result, 7-phenoxy-*N*-(prop-2-yn-1-yl)-1,2,3,4-tetrahydroacridin-9-amine hydrochloride (**15**) has been identified as a permeable agent that shows a balanced pharmacological profile [IC_50_ (*h*AChE) = 1.472 ± 0.024 µM; IC_50_ (*h*BChE) = 0.659 ± 0.077 µM; IC_50_ (*h*MAO-B) = 40.39 ± 5.98 nM], and consequently, as a new hit-ligand that deserves further investigation, in particular *in vivo* analyses, as the preliminary cell viability test results reported here suggest that this is a relatively safe therapeutic agent.

## Graphical Abstract

**Figure:**
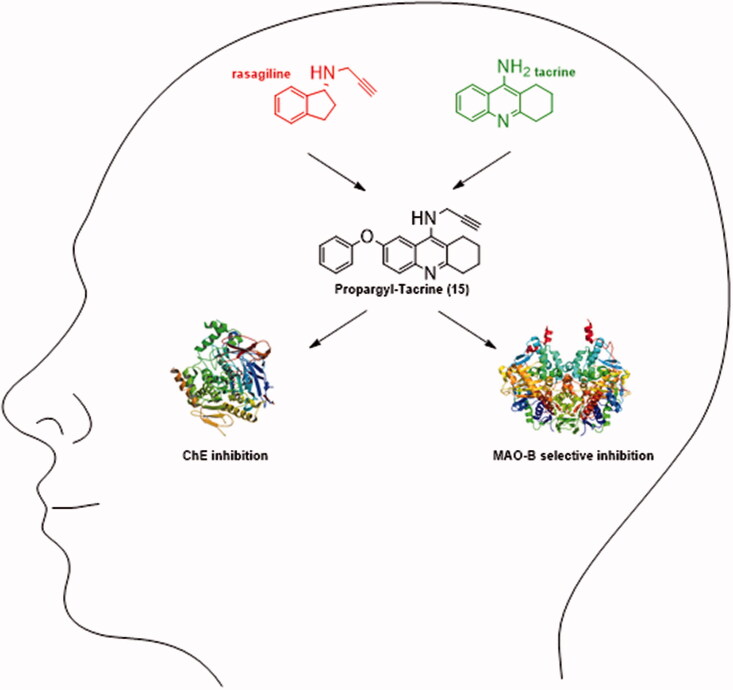

## Introduction

Alzheimer’s disease (AD), the most common cause of dementia, is a growing health concern with huge implications for individuals and society. Current estimates suggest that 44 million people with dementia live worldwide. This number is predicted to increase more than triple by 2050 as the population ages, whereas no effective causal therapeutics are available^1^. The most typical clinical manifestation of AD in the elderly represents insidious and progressive problems associated with episodic memory. The distinctive features of Alzheimer’s pathology are amyloid plaques and neurofibrillary tangles. Amyloid plaques are extracellular accumulations composed of abnormally folded amyloid protein, whereas neurofibrillary tangles are intracellularly lodged paired helical filaments consisting of hyperphosphorylated tau protein. The downstream consequences of these pathological processes include neurodegeneration with synaptic and neuronal loss leading to macroscopic atrophy^2^.

At present, only two classes of therapeutics are available for patients with AD. Administration of cholinesterase (ChE) inhibitors such as donepezil, rivastigmine, and galantamine, is recommended for patients with mild, moderate, or severe AD dementia^3^. Memantine acting as both a non-competitive *N*-methyl-D-aspartate receptor antagonist and a dopamine D_2_ receptor agonist, is approved for use in patients with moderate-to-severe AD^4^. However, such treatment options remain only supportive and symptomatic without attenuating the ultimate prognosis. Medications such as ChE inhibitors and memantine improve memory and alertness, respectively, without changing the life expectancy or overall progression of AD dementia^5^.

Depression is one of the most frequent co-morbid psychiatric disorders in AD^6^. There is consistent evidence that more than 50% of patients with AD suffer from depressive symptoms at some point during the progression of dementia^7^. Regardless of this alarming fact, depression in AD is markedly under-treated. Of all currently available antidepressants, sertraline (selective serotonin reuptake inhibitor) and moclobemide (monoamine oxidase [MAO] inhibitor) show the highest efficacy to treat depression in AD^8^.

Monoamine oxidases (EC 1.4.3.4) catalyse the oxidation of monoamines. These flavoproteins are bound to the outer mitochondrial membrane. In humans, there are two types of MAO: MAO-A and MAO-B. Both isoforms are abundantly present in neurons and glial cells. MAO-A is omnipresent in liver, gastrointestinal tract, or placenta, whereas MAO-B, apart from the central nervous system (CNS), is also produced by blood platelets^9^. The main biological role of MAO-A is the catabolism of neurotransmitters such as serotonin, epinephrine, norepinephrine, and dopamine. The activity of this enzyme increases only slightly with age^10^. On the other hand, MAO-B is responsible for the decomposition of phenylethylamine, benzylamine, and dopamine^11^. The most significant increase in MAO-B concentration is caused by the proliferation of glial cells^10^. Such phenomenon may thus contribute to an excessive reduction of MAO levels in the brain in the elderly. Moreover, it has been confirmed that the activity of MAO increases with the progression of AD. Within the process of amines oxidation, MAO produces aldehydes, ammonia, and hydrogen peroxide. It is particularly hydrogen peroxide that evokes the development of neuronal oxidative stress by disrupting mitochondria. Excessive MAO activation is also responsible for an increase in *β*- and *γ*-secretase expression^10^. Thus, not surprisingly, MAO inhibitors have been considered promising and attractive targets for the therapy of neurodegenerative diseases^12^^,^^13^.

## Design

**Chart 1.**
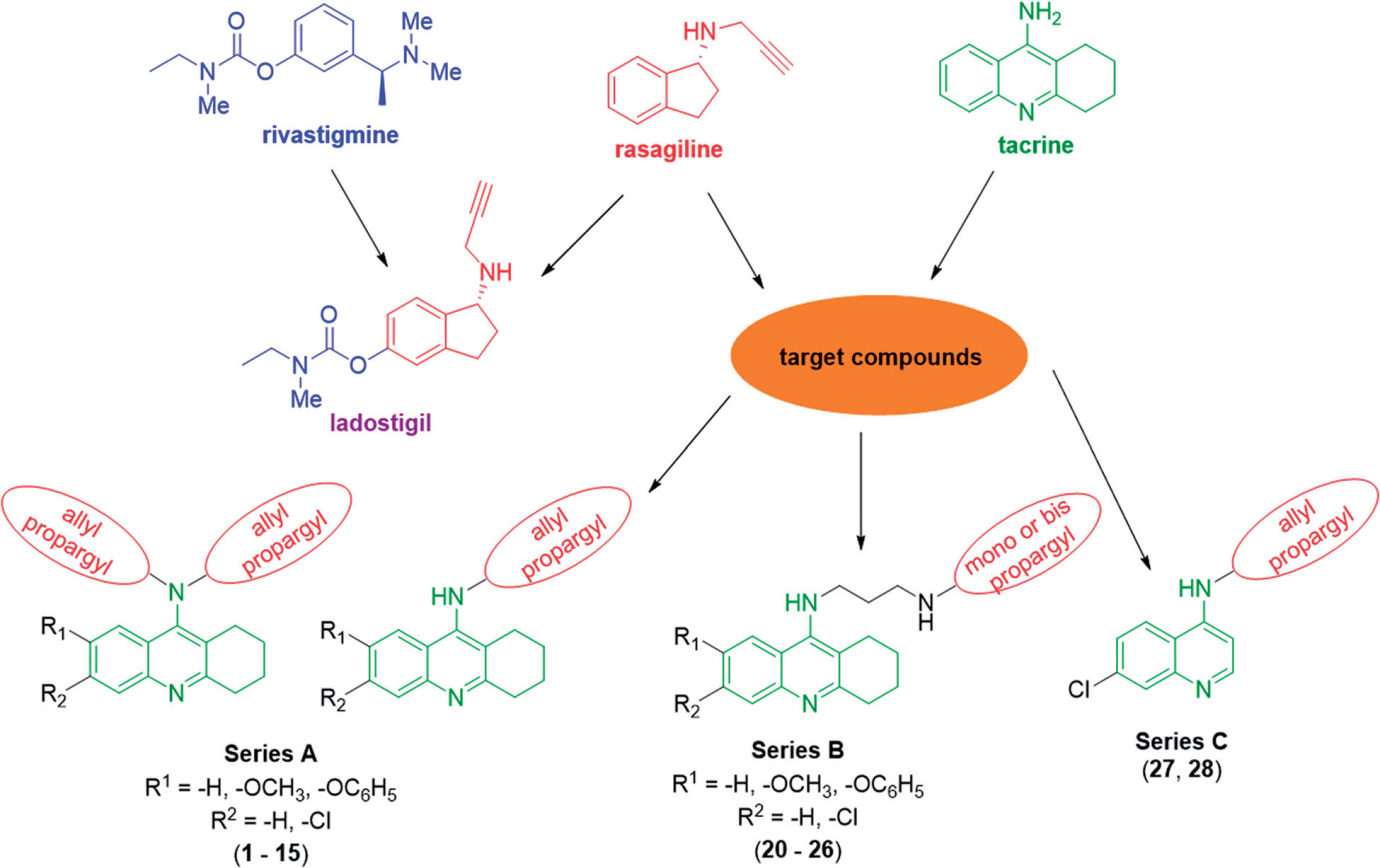
Design strategy of merged propargyl–tacrine ligands.

In our work we have been inspired by ladostigil (TV3326; *N*-propargyl-((3*R*)-aminoindan-5-yl)-ethyl methyl carbamate; Chart 1), a dual cholinesterase and brain-selective MAO-A and MAO-B inhibitor intended for the treatment of dementia co-morbid with extrapyramidal disorders and depression^14^. The rational design of this multipotent molecule is based on the combination of carbamate ChE inhibitory moiety of anti-AD drug rivastigmine (Chart 1) and *N*-propargyl scaffold of rasagiline (Chart 1), an anti-Parkinsonian drug and irreversible selective MAO-B inhibitor^15^. In rodents, oral administration of ladostigil has shown to inhibit brain ChE by 25–40% and antagonise scopolamine-induced spatial memory impairments, pointing out that it is able to penetrate the blood–brain barrier (BBB) sufficiently^16^. Hydrolysis of ladostigil carbamate moiety by pseudo-irreversible inhibition of ChE yields 6-hydroxyrasagiline, to which higher affinity towards both isoforms of MAO is attributed, comparing to non-hydrolyzed ladostigil^17^. Thus, it is most likely that an adequate concentration of 6-hydroxyrasagiline and other metabolites of ladostigil significantly inhibit MAO-A and MAO-B, increasing hereby the levels of dopamine, serotonin, and noradrenaline, which accounts for the respective anti-Parkinsonian and antidepressant properties of ladostigil^18^. In several studies, ladostigil has been shown to possess a broad scale of neuroprotective activities against a variety of neurotoxins and neuronal cell culture models of neurodegeneration^17^^,^^19^.

As an optimal strategy for the design of our compounds, a merged type of multi-target-directed ligands (MTDLs) has been selected since medicinal chemists all over the world aspire to maximise the degree of framework overlap to design as simplest and smallest molecules as possible with favourable physicochemical properties. In merged MTDLs the frameworks are integrated by the use of commonalities in the structures of the building blocks^20^. In our case, rasagiline (Chart 1) and tacrine (THA; Chart 1) have been selected as such building blocks.

Rasagiline is an irreversible inhibitor of MAO-B used as a monotherapy to treat symptoms of early Parkinson’s disease (PD) or as an adjunct therapy in more advanced cases of PD^21^. Findings of structure–activity relationship (SAR) studies of rasagiline provided an evidence that particularly *N*-propargyl moiety is responsible for the promotion of neuronal survival, highlighting its importance in the design of our compounds^22^. However, sequential studies revealed that rasagiline’s neuroprotective activity is not dependent on MAO-B inhibition *via* the interaction of *N*-propargyl moiety with FAD co-factor of the enzyme, but on the ability of rasagiline to regulate the non-amyloidogenic processing of amyloid precursor protein^23^^,^^24^. Apart from *N*-propargyl moiety of rasagiline (**8 − 15**; Chart 1), we have also utilised *N*-allyl motif (**1 − 7**; Chart 1) for comparative purposes.

THA (Chart 1) is a non-selective ChE inhibitor that was approved in the 1990s as the first drug in AD therapy. Despite its high clinical efficiency, THA was withdrawn due to drawbacks associated with hepatotoxicity^25^. However, in view of its easy commercial accessibility, drug-like properties, low molecular weight and, in particular, awareness that THA structure modification may lead to the reduction of adverse effects, it is still widely used by medicinal chemists as a lead scaffold. For designing of our merged structures, we have decided to use not only THA itself, but also 6-chlorotacrine (moiety with higher potential to acetylcholinesterase (AChE) comparing to THA; Chart 1), 7-methoxytacrine (7-MEOTA), scaffold with safer pharmacological profile than THA; Chart 1) and 7-phenoxytacrine (7-PhOTHA), motif with proven dual action towards ChE and *N*-methyl-D-aspartate receptors^26–28^. Apart from simple alkylation of variously modified THA scaffolds, we have decided to synthesise compounds with inserted propylene linker (**20 − 26**; Chart 1) to improve the anticholinesterase activity of mentioned compounds. Finally, compounds **27** and **28** (Chart 1) were synthesised to find out whether quinoline moiety is able to inhibit ChE efficiently as well.

Similar compounds have already been synthesised by Mao et al.^29^ Unlike our group, they concentrated their efforts only on more potent ChE inhibitors. Therefore, they did not evaluate their compounds from the point of view of inhibition of MAOs.

## Chemistry

**Scheme 1a.**
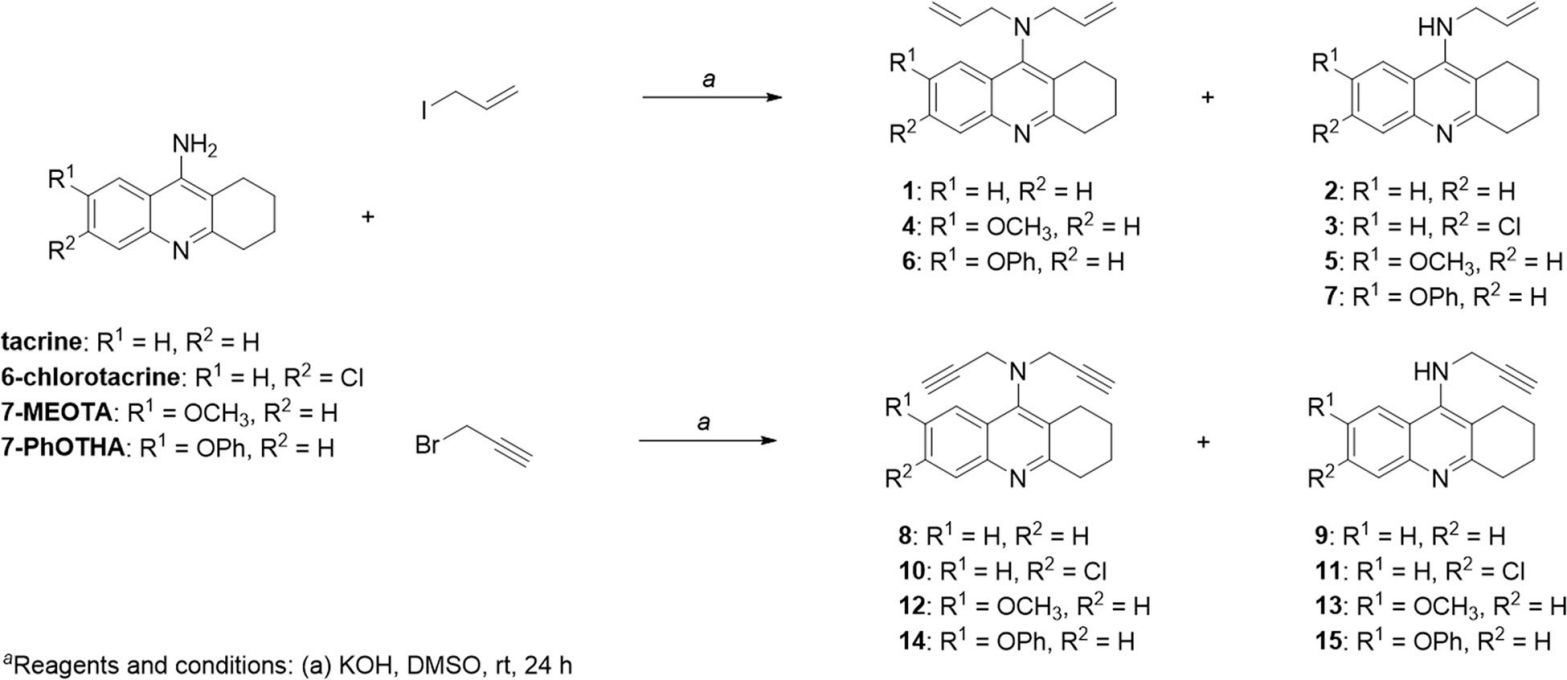
Synthetic strategy resulting in preparation of allylated and propargylated THA derivatives (series A).

General synthetic approaches have been designed for three series of ligands. For series A (Chart 1), the synthetic pathway, outlined in Scheme 1, was initiated by synthesis of THA, 6-chlorotacrine, 7-MEOTA, and 7-PhOTHA following the procedures that have been already published^26^^,^^27^^,^^30^^,^^31^. Afterwards, obtained intermediates reacted with allyl iodide or propargyl bromide in the presence of potassium hydroxide (KOH) in dimethyl sulfoxide (DMSO). By this reaction, two target compounds, i.e. mono- and bis-alkylated THA derivatives, were isolated. An exception was allylation of 6-chlorotacrine, in which just one product, the mono-alkylated compound, was obtained.

**Scheme 2b.**
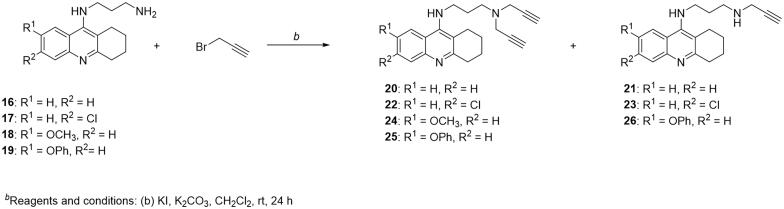
Synthesis of propargylated THA-like compounds with inserted propylene chain (series B).

Series B (Chart 1) consisted of propargylated THA-like congeners with inserted propylene chain between two amino groups. The initial step of the synthetic way included the preparation of intermediates **16 − 19** according to the previous reports (Scheme 2) ^32–34^. Subsequent alkylation of primary amino group by propargyl bromide in the presence of potassium carbonate (K_2_CO_3_) and potassium iodide (KI) in dichloromethane (CH_2_Cl_2_) afforded target products. Similarly, as in the previous pathway, such reaction resulted in the isolation of mono- and bis-alkylated products. However, within this synthetic route, only in case of THA, 6-chlorotacrine and 7-PhOTHA such “two final products in one reaction” phenomenon was observed.

**Scheme 3c.**
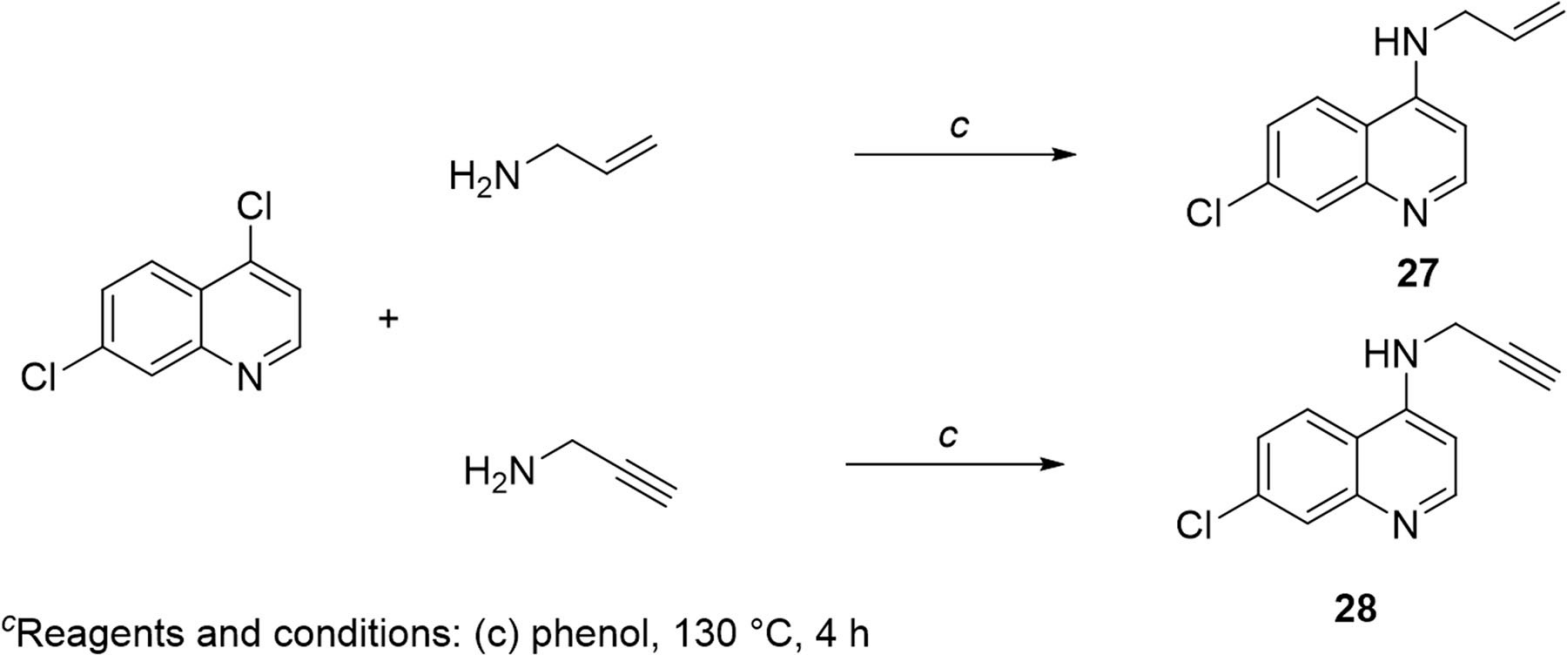
Synthetic approach leading to allylated (**27**) and propargylated (**28**) 7-chloroquinolines (series C).

Finally, to evaluate whether allylated or propargylated 6-chlorotacrine fragments are also active towards ChE and MAO, compounds **27** and **28**, respectively, were synthesised (series C, Chart 1). For this set, 4,7-dichloroquinoline was used as a starting material which was further aminated by allylamine or propargylamine in phenol. The latter synthetic route is depicted in Scheme 3.

All the targeted final tacrines were converted into the corresponding hydrochloride or dihydrochloride salts, prior to their chemical characterisation. All new compounds showed analytical and spectroscopic data in good agreement with their structure (Experimental Section), and were forwarded for biological evaluation. Only compounds **9**, **10,** and **11** (Scheme 1) have been previously described in the literature, and their spectroscopic data are in full accord with ours^29^.

## AChE and BChE inhibitory activity

All synthesised compounds (**1 − 15**, **20 − 28**) were evaluated *in vitro* for their inhibitory activities towards human AChE (*h*AChE; EC 3.1.1.7) and human butyrylcholinesterase (*h*BChE; EC 3.1.1.8). For that purpose, Ellman’s colorimetric assay was used^35^. Further, SAR has been deduced from the data listed in Table 1, where the inhibitory activities of studied compounds are expressed as IC_50_ values, i.e. concentration causing 50% inhibition of the enzymatic activity ± STD. Parent compounds – THA, 6-chlorotacrine, 7-MEOTA, and 7-PhOTHA – were used as reference compounds.

**Table 1.** Inhibitory properties of target and reference compounds towards both *h*ChEs.

Compound	IC_50_ *h*AChE ± STD (µM)^a^	IC_50_ *h*BChE ± STD (µM)^a^	SI (*h*BChE/*h*AChE)^b^
**1**	0.493 ± 0.012	0.716 ± 0.035	1.45
**2**	0.303 ± 0.004	0.488 ± 0.019	1.61
**3**	0.070 ± 0.001	3.219 ± 0.129	31.70
**4**	3.688 ± 0.113	8.758 ± 0.292	2.37
**5**	2.271 ± 0.062	12.53 ± 0.387	5.52
**6**	9.545 ± 0.138	13.19 ± 0.408	1.38
**7**	1.122 ± 0.021	2.768 ± 0.133	2.47
**8**	0.186 ± 0.005	0.937 ± 0.043	5.04
**9**	0.094 ± 0.0008	0.162 ± 0.008	1.72
**10**	0.105 ± 0.004	10.87 ± 1.02	103.52
**11**	0.031 ± 0.003	1.088 ± 0.063	35.10
**12**	5.125 ± 0.184	7.971 ± 0.300	1.56
**13**	1.363 ± 0.039	3.755 ± 0.210	2.75
**14**	2.878 ± 0.084	12.17 ± 0.713	4.23
**15**	1.472 ± 0.024	0.659 ± 0.077	0.45
**20**	0.198 ± 0.024	0.078 ± 0.003	0.39
**21**	0.095 ± 0.001	0.093 ± 0.003	0.98
**22**	0.061 ± 0.002	0.170 ± 0.013	2.79
**23**	0.021 ± 0.002	0.500 ± 0.016	23.81
**24**	1.948 ± 0.046	1.039 ± 0.060	0.53
**25**	2.212 ± 0.074	1.146 ± 0.050	0.52
**26**	1.041 ± 0.040	1.06 ± 0.062	1.02
**27**	10.73 ± 0.406	66.33 ± 2.065	6.18
**28**	8.715 ± 0.242	n.d.	n.d.
**THA**	0.192 ± 0.015	0.086 ± 0.003	0.45
**6-Chlorotacrine**	0.059 ± 0.002	1.698 ± 0.070	28.78
**7-MEOTA**	3.162 ± 0.100	10.72 ± 0.582	3.39
**7-PhOTHA**	0.445 ± 0.045	3.705 ± 0.197	8.33

^a^Results are expressed as the mean of at least three experiments. ^b^Selectivity for *h*AChE is determined as ratio IC_50_(*h*BChE)/IC_50_(*h*AChE). n.d.: not determined.

Regarding *h*AChE, no significant difference was found between the allylated and propargylated analogues in series A (Chart 1). In case of *h*BChE, the situation was slightly divergent, pointing out to more favourable pharmacological effect of propargylated derivatives. Comparison of mono- and bis-analogues of allylated and propargylated THAs of series A revealed that compounds with only one alkyl group led to a drop in activity of both ChEs more noticeably. This phenomenon may be related to hindrance issues of bis-analogues into the enzyme cavities. With respect to the THA fragment and modifications within all series, no surprising findings were concluded, i.e. for *h*AChE, compounds bearing the 6-chlorotacrine skeleton were among the most potent representatives of series, whereas for *h*BChE, the most active compounds were those with unsubstituted THA moiety. A similar pattern can also be observed in the parent compounds. In series B, insertion of the propylene chain led to the desired result, i.e. enhancement of the inhibitory activity of all tested compounds towards both ChEs. Moreover, it should also be mentioned that almost all (except **25** and **26**) derivatives of series B exerted better results than the corresponding parent compounds. Study of the effect of bis-propargyl and monopropargyl group on anti-ChE activity within this series pointed out to more pronounced beneficial effect on *h*AChE inhibition than *h*BChE, which may be explained by the conformational differences between these enzymes. Since the active site of BChE is wider than that of AChE, BChE can thus accommodate the inhibitors with branched linkers^36^. Looking at the results of series C (Chart 1), it is obvious that the absence of tetrahydrobenzene ring leads to a sharp decrease of the inhibitory potential towards both ChEs. Thus, compounds **27** and **28** could be considered as the least active representatives of the whole subset.

When compared with the reference drug THA, only compounds **3**, **8**, **9**, **10**, **11**, **21**, **22**, **23** turned out to be more potent AChE inhibitors than the standard, highlighting propargyltacrine **23** (IC_50_=21 nM) as absolutely the most active towards the mentioned enzyme. With regard to *h*BChE, as "better than standard compounds" could be indicated only derivative **20** (IC_50_ = 78 nM), which could exert a more prominent role at later stages of AD. However, the most balanced ChE inhibitor was ligand **21** showing equipotent and significant values on both ChEs (*h*AChE: IC_50_=0.095 ± 0.001 µM; *h*BChE: IC_50_=0.093 ± 0.003 µM) (Table 1).

Since the biological role of AChE is clear, the exact role of BChE in the organism still has not been completely elucidated. Whereas AChE undergoes a significant reduction within the progression of AD, BChE levels and activity in certain brain regions associated with AD have been shown to increase^37^^,^^38^. Therefore, selective BChE inhibitors could be more effective in patients with advanced stages of the disease. For this purpose, the selectivity indexes (SI) for BChE over AChE have been determined. The highest selectivity for BChE was observed for compounds **15**, **20**, **24,** and **25** with SI ranging from 0.39 to 0.45.

It is interesting to note that previously investigated ligands **9**, **10,** and **11** (Scheme 1) showed different IC_50_ values to those described here, although not comparable, as the used enzymes (*Ee*AChE, *eq*BChE) were not the same^29^.

## Kinetic characterisation of AChE inhibition

**Figure 1.**
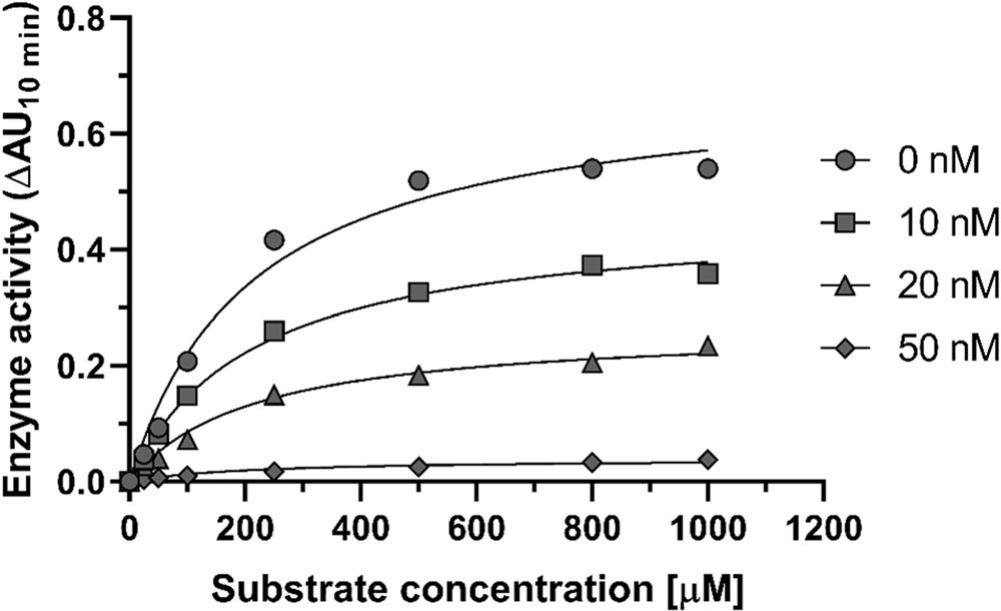
Kinetic study on the mechanism of *h*AChE inhibition by compound **23**.

To gain further insight into the mechanism of *h*AChE inhibition, an enzyme kinetic study was performed on the most potent AChE inhibitor of the series (**23**). The graphical presentation of the steady-state inhibition data of compound **23** for *h*AChE is demonstrated in Figure 1. The analysis of the direct plots revealed a reduction of *V*_max_, whereas *K*_m_ remained unchanged. These findings are consistent with a non-competitive mode of enzyme inhibition. In case of AChE, it means that the preferential binding site of propargyltacrines is the peripheral anionic site (PAS). From the perspective of AD therapy, this is a highly desirable effect since aggregation of amyloid-beta protein (A*β*) and subsequent neurotoxic cascade are catalysed particularly by the PAS of AChE^39^. Replots of the slope versus concentration of **23** gave an estimate of the competitive inhibition constant (*K*_i_) of 12.39 ± 1.40 nM, which is consistent with the IC_50_ (*h*AChE) value obtained above.

## *In vitro* inhibition of MAO-A and MAO-B

To confirm the multipotent biological profile of target compounds **1 − 15**, **20 − 28**, the inhibitory activity towards both isoforms of *h*MAO was determined *in vitro*. Gained results are listed in Table 2 as the residual activity of the enzyme after inhibition by the studied compound at two concentrations (10 and 1 μM). Parent compounds – THA, 6-chlorotacrine, 7-MEOTAand 7-PhOTHA – were evaluated as well. Clorgyline and pargyline were used as standards for *h*MAO-A and *h*MAO-B inhibition, respectively.

**Table 2.** Inhibitory activity of target and reference compounds against both isoforms of *h*MAOs.

Compound	% of MAO-A activity (10 µM)^a^	% of MAO-A activity (1 µM)^a^	% of MAO-B activity (10 µM)^a^	% of MAO-B activity (1 µM)^a^	IC_50_ * h*MAO-B ± STD (nM)^a^
**1**	85	95	62	89	
**2**	58	78	72	48	
**3**	73	76	64	71	
**4**	107	103	61	83	
**5**	62	97	70	82	
**6**	78	76	4	16	
**7**	90	99	2	5	162.65 ± 17.35
**8**	111	103	47	59	
**9**	56	94	61	83	
**10**	92	92	141	106	
**11**	39	89	185	95	
**12**	114	123	45	63	
**13**	82	96	62	74	
**14**	47	93	14	73	
**15**	51	41	3	8	40.39 ± 5.98
**20**	33	78	96	148	
**21**	54	47	90	88	
**22**	102	107	50	87	
**23**	98	104	64	91	
**24**	70	60	82	169	
**25**	80	96	1	6	169.95 ± 7.75
**26**	79	88	4	14	
**27**	19	54	127	150	
**28**	31	39	61	150	
**THA**	60	88	78	78	
**6-Chlorotacrine**	48	61	84	80	
**7-MEOTA**	42	41	76	78	
**7-PhOTHA**	86	99	n.d.	4	22.6 ± 1.6
**Clorgyline**	n.d.	43	n.d.	n.d.	
**Pargyline**	n.d.	n.d.	n.d.	n.d.	80 ± 1

^a^Results are expressed as the mean of at least three experiments. n.d.: not determined.

The data in Table 2 show that the majority of target compounds exhibited low to moderate inhibitory activity towards both isoforms of MAO, with some excellent hints. SAR analysis revealed that the series with 7-phenoxy moiety at tacrine core, irrespective if it was allyl or propargyl derivative, could be highlighted as selective MAO-B inhibitors with excellent inhibitory properties at both tested concentrations. In particular, derivatives **7** (5% of residual MAO-B activity at 1 μM concentration), **25** (6% of residual MAO-B activity at 1 μM concentration) and **15** (8% of residual MAO-B activity at 1 μM concentration) can be classified as the best agents in the series. Therefore, all these compounds, including 7-PhOTHA, were also forwarded for determination of the IC_50_ values towards MAO-B. For comparative purposes, the IC_50_ value of pargyline (a selective MAO-B inhibitor) was determined as well. The data in Table 2 highlight target compound **15** (IC_50_=40 nM) as the most potent MAO-B inhibitor of the series and starting compound 7-PhOTHA (IC_50_=23 nM), being two- and almost four-times as active as the reference compound pargyline (IC_50_=80 nM).

On account of ChE and MAO inhibitory activity results shown in Tables 1 and 2, compounds **7**, **15**, **20**, **21**, **23**, and **25** exhibiting the most balanced pharmacological profile have been selected as MTDLs worthy of further investigation.

## Computational chemistry studies

Binding modes and affinities of **23** to *h*AChE and **15** to *h*MAO-B were estimated by various computational chemistry tools available in Schrodinger 2021–4 employing a Linux-based supercomputer Karolina. As models of *h*AChE and *h*MAO-B, three X-ray structures, available in the online protein databank (rcsb.org) were selected for each enzyme (*h*MAO-B PDB IDs: 2V5Z, 3PO7, 4CRT, *h*AChE PDB IDs: 4EY7, 4M0E, and 7RB6) to account for structural variabilities of the enzymes. All the chosen X-ray models were determined from recombinantly prepared human enzymes, which correspond well with the recombinant enzyme isoforms used in the performed *in vitro* experiments. The main difference between the used X-ray enzyme models consists in the presence of various inhibitors. Using the co-crystalised inhibitors as the enzyme active site locators, the studied ligands **23** and **15** were docked into all six enzyme models with the induced fit docking (IFD) utility on the extended precision level (XP), allowing conformational flexibility to all residues within a cubic gridbox with the edge of 30 Å. The top-scored binding modes resulting from IFD were further analysed by hybrid quantum mechanics/molecular mechanics (QM/MM) calculations, which additionally optimised the molecular systems and provided the potential energies of the enzyme, the ligand and the complex. The tested ligands **15**, **23**, the FAD co-factor, *h*AChE residues: Asp74, Trp86, Trp286, His447, *h*MAO-B residues: Leu171, Ile198, Ile199, Tyr326 were assigned into the QM region and the remaining protein chains into the MM region. These side chain residues were selected as a compromise for the QM simulations due to their importance for the enzymes’ catalytic activities and key interactions with known inhibitors^40^^,^^41^. Combining the force field OPLS_2005 for the MM region and the DFT M06-2X/CC-PVTZ(-F)++ for the QM part, which is actually one of the best functional for analysing non-covalent interactions, it was possible to evaluate the *in silico* binding energy of the selected ligands on a higher level of theory^42^. Finally, the formation of the covalent binding of **15** in *h*MAO-B between the terminal *sp* carbon atom of the *N-*propargyl moiety of **15** and the *sp^2^* nitrogen (i.e. N5) atom of the flavin moiety within the ligand-protein complex was simulated by QM/MM with the same force field and DFT B3LYP/LAV3P++** method, utilising the relaxed scan technique available in QSite & Jaguar 11.4 of Schrodinger 2021–4. In all QM/MM simulations, the interactions between the MM part and the wavefunction of the QM region involved complete electrostatic and van der Waals effects without any cut-off.

**Figure 2.**
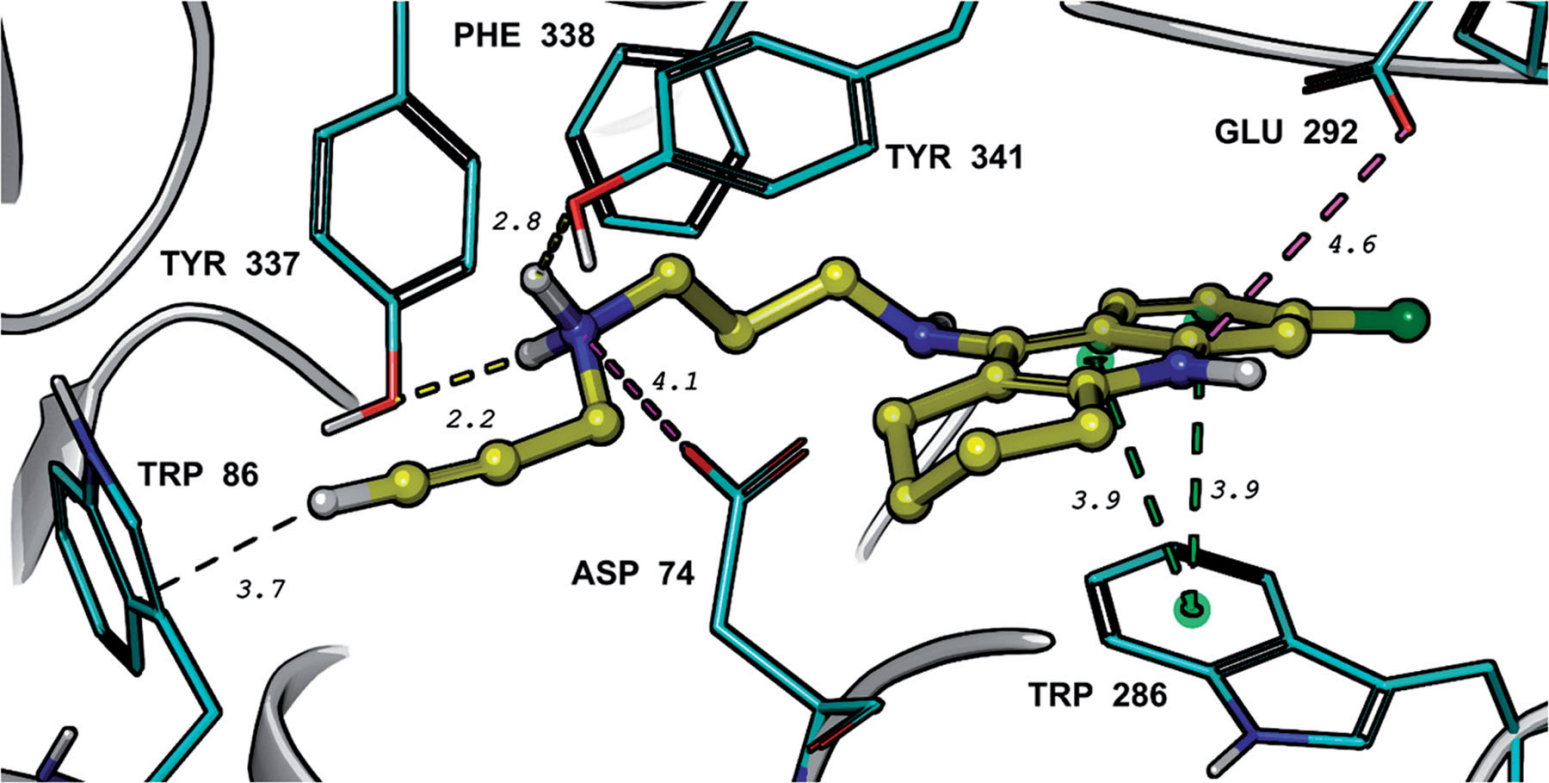
Top-scored binding mode of **23** (yellow) in *h*AChE (PDB ID: 4M0E) determined by IFD and QM/MM (DFT M06-2X/CC-PVTZ(-F)++/OPLS_2005) in Schrodinger 2021–4. The interaction distances (yellow – hydrogen bonds, green – π–π interactions, ping – salt bridge) are given in Å.

The results of IFD in the three models of *h*AChE were different concerning the top-scored binding pose of the 6-chlorotacrine moiety in the enzyme active site. According to the top IFD score, **23** binds in the catalytic active site (CAS) (i.e. in the models 7RB6 and 4EY7), while the top Glide score prioritises the ligand binding in the PAS (i.e. in the model 4M0E). Since the post-docking QM/MM refinement of the binding poses suggests a stronger interaction of the ligand **23** in the PAS (i.e. model with 4M0E), we will consider this binding mode more probable (Supporting Information, Table S1). Such finding is in a strong agreement with the kinetic study realised with compound **23** on *h*AChE, pointing out on non-competitive mode of enzyme inhibition. From the QM/MM simulations, it is evident that **23** strongly binds in the PAS of *h*AChE (PDB ID: 4M0E) forming a cation-π enhanced π–π stacked complex between the 6-chlorotacrine moiety and Tyr286 which is also slightly stabilised from the opposite side with a weak salt bridge between the protonated nitrogen heteroatom of 6-chlorotacrine and Glu292. In addition, the hydrogen atoms on the nitrogen atom bearing the *N-*propargyl function interacted through two hydrogen bonds with Tyr337 and Tyr341. The *N-*propargyl moiety itself exhibited no important interactions with the *h*AChE active site residues and pointed out roughly towards Trp86. All these interactions occurred within a radius of 2.2–4.6 Å from the ligand **23** and, as such, they explain strong ligand affinity for *h*AChE which was observed experimentally (Figure 2).

**Figure 3.**
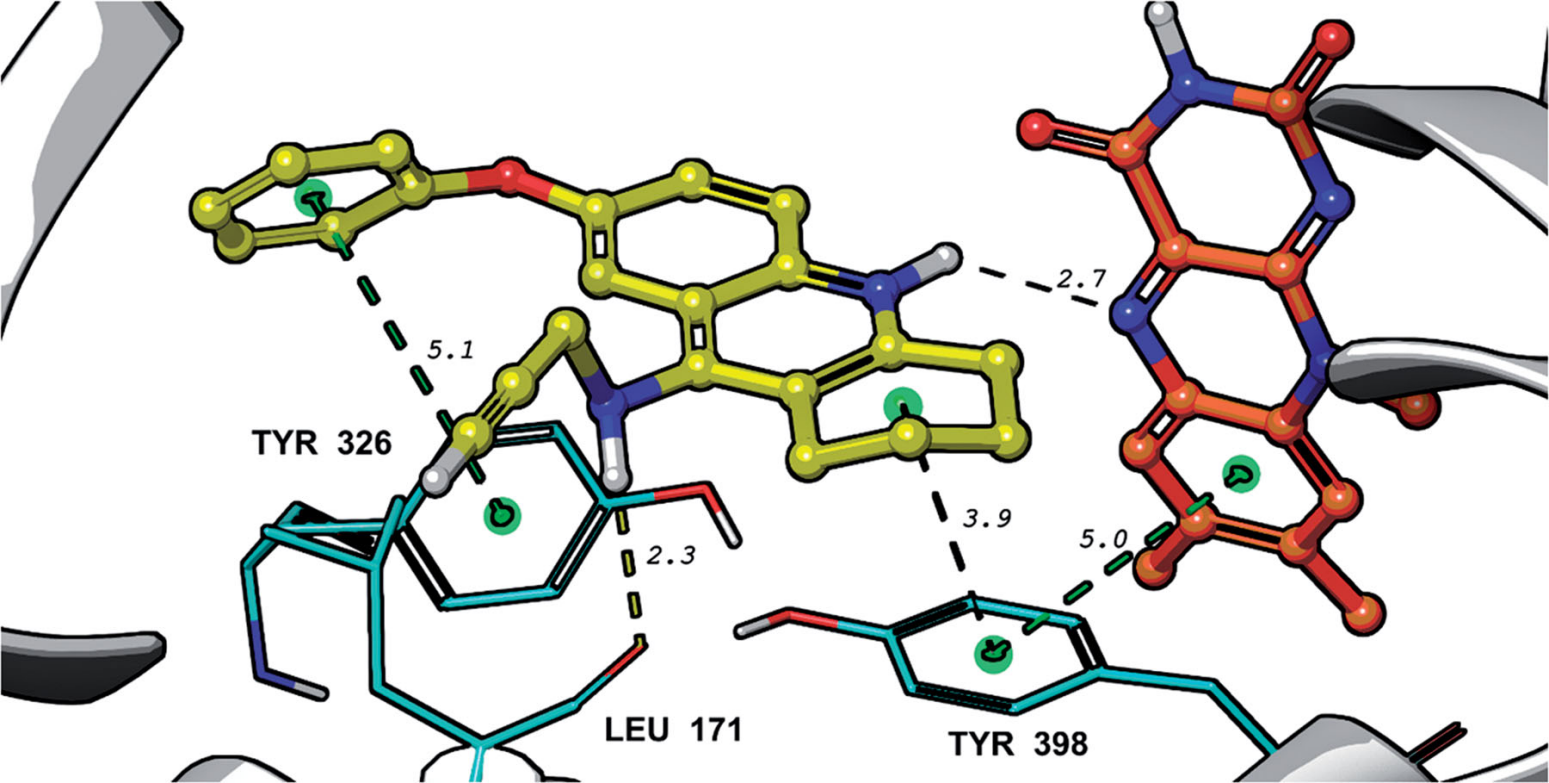
Top-scored binding mode of **15** (yellow) in *h*MAO-B (PDB ID:3PO7) determined by IFD and QM/MM (DFT M06-2X/CC-PVTZ(-F)++/OPLS_2005) in Schrodinger 2021–4. Only the flavine part of the co-factor FAD (red) was displayed, the rest of the molecule was hidden in the figure. The interaction distances (green – π–π interactions, black – auxiliary measurements) are given in Å.

IFD of **15** in the three different *h*MAO-B models provided more similar results to one another than the calculations with *h*AChE. In all *h*MAO-B models used, the tacrine moiety of the ligand **15** was found close to the FAD co-factor while the *N-*propargyl terminus was oriented roughly towards the entrance of the enzyme active site. Preferring the post-docking QM/MM refined results as relatively more reliable, the optimum binding mode of **15** was attributed to the model in *h*MAO-B 3PO7 (Supporting Information, Table S2). Here, the tacrine moiety of the ligand **15** was facing to Tyr398 in a coplanar position exposing the hydrogen atom on the nitrogen heteroatom in a T-shaped conformation to N5 of the FAD co-factor. Additional energetical contribution strengthening the binding mode was provided by a weak π-π interaction of the phenoxy function of **15** with Tyr326 and by a hydrogen bond between Leu171 and the hydrogen attached to the secondary amino group of **15** (Figure 3). Given the predicted score values and potential energies, **15** seems to be a weaker inhibitor of *h*MAO-B than **23** of *h*AChE (Supporting Information, Tables S1 and S2). Although the results of IFD and QM/MM simulations represent only gas phase interactions in the global potential energy minimum, it is very probable that **15** binds in *h*MAO-B rather weakly. Nonetheless, the inhibition potency of **15** could be significantly enhanced by covalent binding of the *N-*propargyl function to the FAD co-factor.

To estimate the possibility of covalent binding between the *N-*propargyl terminal carbon atom of **15** (i.e. C(*sp*)) and N5 of the FDA co-factor (i.e. N5(*sp^2^*), the QM/MM optimised binding mode of **15** in *h*MAO-B 2V5Z was selected for additional computational studies due to relatively suitable arrangement of the reaction atoms. The relaxed scan protocol based on QM/MM calculations revealed that the *N-*propargyl moiety can relatively easily rotate approximately by 120° around the single bond with the secondary amino group of **15** in the enzyme active site until it is directed to the N5 of FAD. Further shortening of the C(*sp*)-N5(*sp^2^*) distance during the relaxed scan shifted the system over a slight potential barrier and stabilised it at *r* = 6 Å where the terminal hydrogen of the *N-*propargyl function was directed to the oxygen atom on C4 of **15** (2.4 Å), forming a non-covalent hydrogen bond-like interaction. In this reaction coordinate point, the tacrine remained approximately in the same T-shaped orientation to the FAD co-factor as it was found in the starting conformation. Next scanning steps increased the total energy of the molecular system until the C(*sp*)-N5(*sp^2^*) distance reached *r* = 2.0 Å, which could be considered as a rough estimate of the transition state. Similarly to the study by Borstnar et al. ^43^, our QM/MM simulation predicted a stable cycloaddition between the alkyne part of **15** and the N5-C4a-C4-O diene part of the FAD co-factor. Unfortunately, the predicted relative activation energy of the cycloaddition is about 87 kcal/mol, relatively to the starting conformation, and the produced endoergic cycloadduct has approximately by 40 kcal/mol (*r* = 1.5 Å) higher potential energy than that of the initial state on the reaction coordinate (Supporting Information, Figure S1). Thus, the cycloaddition of **15** to the FAD seems to be improbable under given experimental conditions in the *h*MAO-B 2V56 model. Analogical QM/MM simulations starting from a different conformation of **15** found in *h*MAO-B 4CRT QM/MM refined docking revealed that the terminal carbon atom C(*sp*) of the *N-*propargyl moiety can bind with N5 atom in FAD with the activation energy of roughly 29 kcal/mol with respect to the energetical minimum on the reaction coordinate (*r* = 6 Å), but the product still had a relatively high potential energy (30 kcal/mol) thanks to the formation of carbanion at the proximal end of the triple bond in **15**. Importantly, the QM/MM relaxed scan technique led to a significant energy decrease of the starting conformation in the course of the simulation, but this effect is artificial due to setting the starting point of the reaction coordinate out of the global energy minimum on the potential energy surface (Supporting Information, Figure S2). Thus, it is more appropriate to derive the activation energy estimate with respect to the above-mentioned energetical minimum on the reaction coordinate (*r* = 6 Å). In all QM/MM simulations, we did not observe any spontaneous stabilisation of the product by rearrangement, but a more advanced QM/MM setting with explicit water molecules could likely promote protonation of the carbanion and sufficient stabilisation of the supposed adduct of **15** and the FAD co-factor. Such calculations with solvated active enzyme site would require significantly higher computational power. Nonetheless, the performed QM/MM simulations in *h*MAO-B 4CRT suggest that a single covalent binding of **15** to the FAD of *h*MAO-B is not unreasonable to expect. Finally, it is worth mentioning that the top-scored binding mode of **15** obtained in *h*MAO-B 3PO7 by docking was also analysed by the same QM/MM simulation of covalent binding, but the relaxed scanning could not be completed due to induced cleavage of the *N*-propargyl function from the rest of the ligand molecule.

Following recommendations of one of this study’s reviewer, an additional IFD screening of all compounds (i.e. **1 − 28**, THA, 6-chlolorotacrine, 7-MEOTA, 7-PhOTHA, clorgyline, and pargyline) was performed in models of *h*AChE (PDB ID: 4M0E)*, h*BChE (PDB ID: 6QAC)*, h*MAO-A (PDB ID: 2Z5X), and *h*MAO-B (PDB ID: 3PO7) (Supporting Information, Table S3). From the interactions detected between the tested ligands and the respective enzyme binding site residues, it seems probable that strong and selective inhibition in *h*AChE is supported by formation of a salt-bridge between ligands and Asp74 while weaker *h*AChE inhibitors are bound only with residues such as Phe297, Phe295, or Tyr72 by π–π interactions (Supporting Information, Table S4). In *h*BChE, a strong inhibition is probably enabled by a salt bridge between ligand molecules and Glu197. Ligand-protein interactions with aromatic residues such as Trp82 were observed for strong as well as weak inhibitors and their contribution to inhibition potency towards *h*BChE thus does not seem to be decisive (Supporting Information, Table S5). Based on the predicted binding modes of the tested compounds in *h*MAO-A and *h*MAO-B, the inhibition selectivity for *h*MAO-B may be increased when the ligands can interact simultaneously by a hydrogen bond with the FAD co-factor and by π–π interactions with Tyr326. The selective and strong inhibition potency for both *h*MAO-A/B is probably lost if the ligands are not stabilised in the binding pose by several interactions in different parts of their molecules (e.g. with tacrine moiety, linkers, and phenoxy function) (Supporting Information, Tables S6 and S7). However, the ligand binding poses resulting from the IFD screening should be taken as preliminary information, which has to be further evaluated by advanced molecular dynamics studies to corroborate properly the experimental data (e.g. by metadynamics or Free energy perturbation).

## Prediction of BBB permeability

To predict the ability of selected compounds **7**, **15**, **20**, **21**, **23**, **25** and reference compounds to penetrate through the BBB, the parallel artificial membrane permeability assay (PAMPA) was used, previously described by Di et al.^44^ Such an experiment was also realised on seven commercial drugs whose central un/availability was confirmed *in vivo*^45^^,^^46^. To validate the methodology, reported permeability values of commercial drugs were compared with the experimental data. The results of commercial drugs, as well as synthesised compounds, are summarised in Table 3. Compounds with effective permeability (*Pe*) values lower than 2.0 × 10^−6^ cm s ^− 1^ have been classified as non-BBB permeable (CNS-), while compounds with *Pe* higher than 4.0 × 10^−6^ cm s ^− 1^ have been indicated as BBB permeable (CNS+). Based on obtained results it is obvious, that all selected compounds demonstrated a good probability to cross BBB via passive diffusion and are thus suitable drug candidates worthy of further investigation.

**Table 3.** Prediction of BBB penetration of studied, reference and commercial compounds.

Ligand	*Pe* ± SEM (×10^−6^ cm s^-1^)^a^	CNS predicted availability^b^
**7**	4.22 ± 0.17	CNS+
**15**	10.4 ± 0.94	CNS+
**20**	12.19 ± 1.12	CNS+
**21**	6.07 ± 0.53	CNS+
**23**	6.32 ± 0.88	CNS+
**25**	14.0 ± 1.03	CNS+
THA	6.0 ± 0.6	CNS+
6-Chlorotacrine	11.0 ± 0.5	CNS+
7-MEOTA	17.0 ± 3.6	CNS+
7-PhOTHA	15.9 ± 1.49	CNS+
Donepezil	21.9 ± 2.1	CNS+
Rivastigmine	20.0 ± 2.1	CNS+
Ibuprofen	18.0 ± 4.3	CNS+
Chlorothiazide	1.1 ± 0.5	CNS−
Furosemide	0.2 ± 0.07	CNS−
Ranitidine	0.04 ± 0.02	CNS−
Sulfasalazine	0.09 ± 0.05	CNS−

^a^The results are the mean of at least three independent measurements ± SEM.

*^b^*CNS + (high BBB permeation predicted): *Pe* (×10^−6^ cm s^−1^) > 4.0;.

CNS – (low BBB permeation predicted): *Pe* (×10^−6^ cm s^−1^) < 2.0;

CNS ± (BBB permeation uncertain): *Pe* (×10^−6^ cm s^− 1^) from 4.0 to 2.0^44^.

## *In vitro* neurotoxicity

Since the target organ of proposed hybrids is supposed to be CNS, the neuronal toxicity profile of selected compounds **7**, **15**, **20**, **21**, **23,** and **25** on human neuroblastoma cell line (SH-SY5Y) using the colorimetric 3-(4,5-dimethylthiazol-2-yl)-2,5-diphenyl-tetraziolium bromide (MTT) assay, was determined. The results are presented in Table 4, in terms of mean concentration to cause 50% growth inhibition (IC_50_). THA, 6-chlorotacrine, 7-MEOTA, and 7-PhOTHA were tested as well as reference compounds. Reduction in cell viability of SH-SY5Y was observed for all selected hybrids. Looking at the IC_50_ values of 7-PhOTHA analogues **7**, **15,** and **25** and 6-chlorotacrine derivative **23**, it is evident that they exerted more pronounced ability to decrease the viability of neuronal cells compared to THA. Their toxicity ranged in the same order of magnitude as their parent compounds 7-PhOTHA and 6-chlorotacrine. Although the insertion of the propylene linker in compounds **20** and **21** led to increase in the inhibitory potential towards ChEs comparing THA, in term of *in vitro* neurotoxicity, the effect was quite opposite, i.e. insertion of the side chain caused an increase in cytotoxicity of mentioned compounds. Such phenomenon could be attributed to the higher lipophilicity of the hybrids. Quite interestingly, compound **23**, active towards AChE and ligand **15**, active towards MAO-B at approx. 1 μM concentration, could be considered relatively safe.

**Table 4.** Cytotoxicity profile of tested compounds on the SH-SY5Y and HepG2 cell lines after 24 h incubation.

Compound	IC_50_ ± SEM (µM) SH-SY5Y^a^	IC_50_ ± SEM (µM) HepG2^a^
**7**	12.38 ± 0.44	10.30 ± 0.83
**15**	23.39 ± 2.55	16.94 ± 0.77
**20**	31.35 ± 1.55	38.26 ± 1.75
**21**	38.82 ± 0.75	25.86 ± 0.73
**23**	18.29 ± 0.80	27.65 ± 1.21
**25**	3.75 ± 0.26	5.53 ± 0.14
THA	122.25 ± 1.59	168.47 ± 3.63
6-Chlorotacrine	50.40 ± 1.28	43.20 ± 1.17
7-MEOTA	53.26 ± 0.38	44.37 ± 3.35
7-PhOTHA	18.29 ± 0.18	22.33 ± 0.12

^a^Values are expressed as the mean of triplicate ± SEM.

## Hepatotoxicity assessment

The major issue of clinical use of tacrine is hepatotoxicity which led to its withdrawal from the clinical practice in 2003^47–49^. However, as it was demonstrated in several cases, modification of tacrine structure or its hybridisation could change its toxicity profile leading to safer tacrine derivatives^50^. Owing to the fact that our newly synthesised compounds are tacrine-like derivatives, the cytotoxic effect of selected candidates **7**, **15**, **20**, **21**, **23**, and **25** on human hepatocellular carcinoma cell line (HepG2) was determined. Obtained results (Table 4) were then compared with that of the reference compounds. In general, the hepatotoxic effect of selected compounds showed a very similar pattern as *in vitro* neurotoxicity. Similarly, and as before, compounds **23** and **15** could be considered as relatively non-hepatotoxic agents.

Mao et al. tested neurotoxicity of compounds **9** and **11** (Scheme 1) on the human neuroblastoma cell line, SH-SY5Y, using the colorimetric MTT assay as we did, and found that “**9** had nearly no effect on the viability of SH-SY5Y cells at the concentrations of 10, 50 and 100 µM, which is a lower cytotoxicity than THA, whereas compound **11** and THA had similar effects on the viability of SH-SY5Y cells at all of the tested concentrations” ^29^. Similarly, Mao et al. tested hepatotoxicity of compounds **9** and **11** (Scheme 1) on human hepatic stellate cells using similar method as we did, and found that tacrines **9** and **11 “**exhibited higher cell viability (lower hepatotoxicity) compared with THA. At the concentration of 100 µM, **9** and **11** exhibited nearly no hepatotoxicity while THA had a 59.49% cell survival rate at the same concentration. At a higher concentration of 200 µM, compound **9** still had a 91.38% cell survival rate (THA and **11** gave 48.65% and 57.78%, respectively), which indicated that **9** almost eliminated the hepatotoxicity of THA and is a potential lead compound for the treatment of AD” ^29^. To sum up, their results, unlike ours, pointed out on lower toxic effect of studied compounds **9** and **11** in comparison to THA, but such discrepancies may be attributed to differences in cell lines used^29^.

## Conclusion

Treatment options for AD remain supportive and symptomatic without attenuation of the ultimate prognosis. Medications such ChE inhibitors and memantine improve memory and alertness, respectively, without changing the life expectancy or overall progression of AD dementia. Moreover, depression in AD still remains grossly undertreated, and specific treatments for depression in AD have not been identified.

Thus, the hybrid propargyltacrines proposed here by our group could be a possible mean of how to help those 50% of AD patients who concurrently suffer from the depressive syndrome. Particularly, among all the hybrids investigated here in the diverse pharmacological assays, 7-phenoxy-*N*-(prop-2-yn-1-yl)-1,2,3,4-tetrahydroacridin-9-amine hydrochloride (**15**) (Scheme 1), bearing *N*-propargyl and 7-phenoxytacrine moieties, a permeable agent that shows a balanced pharmacological profile [IC_50_ (*h*AChE) = 1.472 ± 0.024 µM; IC_50_ (*h*BChE) = 0.659 ± 0.077 µM; IC_50_ (*h*MAO-B) = 40.39 ± 5.98 nM], is a new hit-ligand that deserves further investigation. In particular, evaluated, especially *in vivo*, for its safety and therapeutic effect, as the preliminary cell viability test results reported here suggest that compound **15** is relatively safe (IC_50_ [SH-SY5Y] = 23.39 ± 2.55 µM; IC_50_ [HepG2] = 16.94 ± 0.77 µM) (Table 4). Interestingly, note that reference 7-MEOTA, in the same tests, shows better values (IC_50_ (SH-SY5Y) = 53.26 ± 0.38 µM; IC_50_ [HepG2] = 44.37 ± 3.35 µM) than compound **15**, but lower than even THA (IC_50_ [SH-SY5Y] = 122.25 ± 1.59 µM; IC_50_ [HepG2] = 168.47 ± 3.63 µM), is considered a relatively safe THA derivative as proved in many *in vivo* studies^28^. This is why, although the results for hybrid **15** on neuroblastoma and HepG2 were not so convincing as the results on enzymes did, we suppose that chiefly *in vivo* experiments will be able to tell us more about the real toxicological profile of the highlighted drug.

## Experimental section

### Chemistry

#### General chemical methods

All the chemical reagents used were purchased from Sigma-Aldrich (Czech Republic). Solvents for synthesis were obtained from Penta chemicals Co. The solvents and additives used for LC–UV–MS analyses were purchased from Sigma-Aldrich (Czech Republic) in LC–MS grade purity. The course of the reactions was monitored by thin-layer chromatography on aluminium plates precoated with silica gel 60 F254 (Merck, Czech Republic) and then visualised by UV 254. Melting points were determined on a melting point apparatus M-565 (Büchi, Switzerland) and are uncorrected. Uncalibrated purity at the wavelength of 254 nm was ascertained by a LC-UV system Dionex Ultimate 3000 RS which consisted of a binary high-pressure gradient pump HPG-3400RS connected to a vacuum degasser, a heated column compartment TCC-3000, an autosampler WTS-3000 equipped with a 25 μL injection loop and a VWD-3000 ultraviolet detector. As the stationary phase, a Waters Atlantis dC18 100 Å (2.1 × 100 mm/3 µm) column was used along with a protective in-line filter (Vici Jour) and a frit of 0.5 *µ*m pores. The mobile phase was mixed from two components: ultrapure water (MPA) and acetonitrile (MPB), both acidified by 0.1% (*v*/*v*) of formic acid. The studied compounds were first properly dissolved in methanol (c∼0.1 mg/mL) and then analysed by the LC-UV-MS system (MS setting is described below). The following ramp-gradient programme was used for the elution: 0–1 min: 10% MPB, 1–4 min: 10 − 100% MPB linearly, 4–5 min: 100% MPB, 5–7.5 min: 10% MPB. The mobile phase flow-rate in the gradient elution was set to 0.4 mL/min. In the LC-UV analyses, all the synthesised compounds exhibited uncalibrated chromatographic purity 95 − 99 at a wavelength 254 nm. NMR spectra of target compounds were recorded on Varian S500 spectrometer (operating at 500 MHz for ^1^H and 126 MHz for ^13^C; Varian Comp. Palo Alto, CA). Chemical shifts are reported in parts per million (ppm). Spin multiplicities are given as s (singlet), d (doublet), dd (doublet of doublets), t (triplet), q (quartet), p (pentaplet), or m (multiplet). The coupling constants (*J*) are reported in Hertz (Hz). High-resolution mass spectra (HRMS) were determined by Q Exactive Plus hybrid quadrupole-orbitrap spectrometer which was attached to the above-mentioned LC-UV system. Ions for HRMS were generated by a heated electro-spray ionisation source (HESI) working in positive mode under the following settings: sheath gas flow rate 40 arbitrary units (a.u.), aux gas flow rate 10 a.u., sweep gas flow rate 2 a.u., spray voltage 3.2 kV, capillary temperature 350 °C, aux gas temperature 300 °C, S-lens RF level 50, microscans 1, maximal injection time 35 ms, automatic gain control 1e6, resolution of the Fourier transformation 140,000. The applied full-scan MS analyses monitored positive ions within *m/z* range of 100 − 1500. In order to increase the accuracy of HRMS, internal lock-mass calibration was employed utilising polysiloxane traces of *m/z* = 445.12003 ([M + H]^+^, [C_2_H_6_SiO]_6_) present in the mobile phases besides the ordinary MS external calibration system by Pierce™ LTQ ESI Positive Ion Calibration Solution (Sigma-Aldrich, Czech Republic). The chromatograms and HRMS spectra were processed in Chromeleon 6.80 and Xcalibur 3.0.63 software, respectively.

#### General procedure for synthesis of allylated THA derivatives (1–7)

THA hydrochloride, 6-chlorotacrine, 7-methoxytacrine hydrochloride, or 7-phenoxytacrine hydrochloride (1 eq) were dissolved in 20 mL of DMSO. Subsequently grinded KOH (3 eq) was added. In case of 6-chlorotacrine derivatives, only 2 equivalents of grinded KOH were utilised, since 6-chlorotacrine was not in hydrochloride form. Formed suspension was stirred for 2 h at room temperature under inert conditions. Then, allyl iodide (2 eq) was added dropwise to the stirring solution. The resulting mixture was left to stir for additional 24 h under the same conditions. The reaction was diluted with water (100 mL) and extracted four times with ethyl acetate (75 mL). Collected organic layers were dried over Na_2_SO_4_. Excessive solvent was evaporated. Crude product was purified by column chromatography using silica gel pre-treated with triethylamine and hexane/ethyl acetate (1/1) as eluent. Isolated pure bases were dissolved in diethylether and saturated with HCl gas. The solvent was evaporated. Precipitation from MeOH/diethylether gave the final products in the form of hydrochloride salt.

#### General procedure for synthesis of propargylated tacrine derivatives (8–15)

Tacrine hydrochloride, 6-chlorotacrine, 7-methoxytacrine hydrochloride, or 7-phenoxytacrine hydrochloride (1.00 g, 1 eq) were dissolved in 20 mL of DMSO. Subsequently grinded KOH (3 eq) was added. In case of 6-chlorotacrine derivatives, only 2 equivalents of grinded KOH were utilised, since 6-chlorotacrine was not in hydrochloride form. Formed suspension was stirred for 2 h at room temperature under inert conditions. Then, propargyl bromide solution (80%, 2 eq) was added dropwise to the stirring solution. The resulting mixture was left to stir for additional 24 h under the same conditions. The reaction was diluted with water (100 mL) and extracted with ethyl acetate (4 × 75 mL). The organic layers were collected, dried over Na_2_SO_4_ and concentrated *in vacuo*. Crude product was purified by column chromatography using triethylamine-pre-treated silica gel and hexane/ethyl acetate (3/1) as eluent. Isolated pure bases were dissolved in diethylether and saturated with HCl gas. The solvent was evaporated. Precipitation from MeOH/diethylether gave the final products in the form of hydrochloride salt.

#### General procedure for synthesis of propargylated THA-like substances with inserted propylene chain (20–26)

Substituted and unsubstituted *N*-(1,2,3,4-tetrahydroacridin-9-yl)propane-1,3-diamine (1.00 g, 1 eq) was dissolved in CH_2_Cl_2_ (30 mL). Thereafter, K_2_CO_3_ (2 eq) and KI (0.1 eq) were added. Inert gas was introduced into the reaction mixture. Finally, propargyl bromide solution (80%, 1.1 eq) was added dropwise. The reaction was left to stir for 24 h at RT under inert conditions. Subsequently, crude product was filtered and washed with MeOH (30 mL). Filtrate was evaporated and purified by column chromatography using triethylamine-pre-treated silica gel and hexane/ethyl acetate (3/1 → 1/1) as gradient eluent. Isolated pure bases were dissolved in CH_2_Cl_2_ and saturated with HCl gas. Solvent evaporation gave the final products in the form of hydrochloride salt.

#### General procedure for synthesis of allylated (27) and propargylated (28) 7-chloroquinolines

4,7-Dichloroquinoline (1.00 g, 1 eq) was combined with phenol (4.28 g, 9 eq) and heated to 85 °C, until complete dissolution of both reagents. Then, 2 equivalents of allylamine (0.76 mL, 2 eq) or propargylamine (0.65 mL, 2 eq), respectively, were added. The reaction mixture was heated up to 130 °C and left to stir for 2 h. Thereafter, other 2 equivalents of allylamine (0.76 mL, 2 eq) or propargylamine (0.65 mL, 2 eq) were added. The reaction was stirred for additional 2 h. The mixture was cooled to RT. The residue was dissolved in CH_2_Cl_2_ (100 mL) and extracted with 2 M NaOH (100 mL) to remove phenol. Collected organic layers were washed with brine (100 mL) and subsequently with water (100 mL), dried over Na_2_SO_4_ and concentrated *in vacuo*. Crude product was purified by column chromatography using silica gel and hexane/ethyl acetate/triethylamine (3/2/0.1) as eluent. Isolated pure base was dissolved in CH_2_Cl_2_ and saturated with HCl gas. Solvent evaporation gave the final product in the form of hydrochloride salt.

### N,N-Di(prop-2-en-1-yl)-1,2,3,4-tetrahydroacridin-9-amine hydrochloride (1)

Yield 11%. mp 177.3–178.1 °C. Purity: 98%. ^1^H NMR (500 MHz, Methanol-*d*_4_) δ 8.03–7.94 (m, 2H), 7.60–7.55 (m, 1H), 7.45–7.39 (m, 1H), 5.88–5.83 (m, 1H), 5.82–5.78 (m, 1H), 5.19–5.13 (m, 2H), 5.13–5.07 (m, 2H), 3.88 (d, *J* = 6.6, 4H), 3.13 (t, *J* = 6.5 Hz, 2H), 2.90 (t, *J* = 6.4 Hz, 2H), 1.99–1.92 (m, 2H), 1.88–1.82 (m, 2H). ^13^C NMR (126 MHz, Methanol-*d*_4_) δ 160.3, 152.8, 147.9, 135.5, 128.8, 128.4, 128.1, 126.4, 124.8, 124.3, 117.2, 55.5, 33.9, 26.7, 22.9, 22.9. HRMS: [M + H]^+^ 279.1852 (calculated for [C_19_H_23_N_2_]^+^: 279.1861).

### N-(prop-2-en-1-yl)-1,2,3,4-tetrahydroacridin-9-amine hydrochloride (2)

Yield 17%. mp 223.7–224.1 °C. Purity: 99%. ^1^H NMR (500 MHz, Methanol-*d*_4_) δ 8.35 (d, *J* = 8.4 Hz, 1H), 7.86 − 7.75 (m, 2H), 7.57 − 7.49 (m, 1H), 6.21 − 6.10 (m, 1H), 5.42 − 5.30 (m, 2H), 4.60 − 4.50 (m, 2H), 3.03 (t, *J* = 5.9 Hz, 2H), 2.72 (t, *J* = 5.8 Hz, 2H), 2.02 − 1.90 (m, 4H). ^13^C NMR (126 MHz, Methanol-*d*_4_) δ 158.3, 151.7, 139.6, 135.5, 134.0, 126.6, 126.2, 120.0, 117.4, 116.7, 112.9, 50.3, 29.3, 24.8, 22.9, 21.8. HRMS: [M + H]^+^ 239.1538 (calculated for [C_16_H_19_N_2_]^+^: 239.1548).

### 6-Chloro-N-(prop-2-en-1-yl)-1,2,3,4-tetrahydroacridin-9-amine hydrochloride (3)

Yield 48%. mp 176.9–177.5 °C. Purity: 98%. ^1^H NMR (500 MHz, DMSO-*d*_6_) δ 8.33 (d, *J* = 9.2 Hz, 2H), 8.11 (d, *J* = 2.2 Hz, 1H), 7.52 (dd, *J* = 9.3, 2.2 Hz, 1H), 6.14 − 6.02 (m, 1H), 5.30 − 5.19 (m, 2H), 4.54 − 4.40 (m, 2H), 3.02 − 2.96 (m, 2H), 2.65 (t, *J* = 5.6 Hz, 2H), 1.81 (dd, *J* = 6.9, 4.1 Hz, 4H). ^13^C NMR (126 MHz, DMSO-*d*_6_) δ 155.7, 151.2, 138.7, 137.1, 134.9, 127.8, 125.2, 118.0, 116.7, 113.9, 111.7, 45.4, 28.0, 23.9, 21.4, 20.3. HRMS: [M + H]^+^ 273.1148 (calculated for [C_16_H_18_ClN_2_]^+^: 273.1159).

### 7-Methoxy-N,N-di(prop-2-en-1-yl)-1,2,3,4-tetrahydroacridin-9-amine hydrochloride (4)

Yield 10%. mp 168.9–169.8 °C. Purity: 95%. ^1^H NMR (500 MHz, DMSO-*d*_6_) δ 8.29 (d, *J* = 9.2 Hz, 1H), 7.61 (dd, *J* = 9.2, 2.7 Hz, 1H), 7.29 (d, *J* = 2.6 Hz, 1H), 5.90 − 5.85 (m, 1H), 5.85 − 5.80 (m, 1H), 5.30 − 5.23 (m, 2H), 5.23 − 5.18 (m, 2H), 4.06 (d, *J* = 6.5 Hz, 4H), 3.95 (s, 3H), 3.29 − 3.20 (m, 2H), 2.85 − 2.77 (m, 2H), 1.92 − 1.84 (m, 2H), 1.84 − 1.75 (m, 2H). ^13^C NMR (126 MHz, DMSO-*d*_6_) δ 159.7, 158.2, 153.8, 134.8, 133.8, 127.8, 126.4, 125.0, 122.2, 119.2, 104.3, 56.3, 55.2, 28.6, 26.4, 22.0, 20.8. HRMS: [M + H]^+^ 309.1957 (calculated for [C_20_H_25_N_2_O]^+^: 309.1967).

### 7-Methoxy-N-(prop-2-en-1-yl)-1,2,3,4-tetrahydroacridin-9-amine hydrochloride (5)

Yield 41%. mp 242.0–243.5 °C. Purity: 96%. ^1^H NMR (500 MHz, Methanol-*d*_4_) δ 7.74 (d, *J* = 9.2 Hz, 1H), 7.66 (d, *J* = 2.6 Hz, 1H), 7.49 (dd, *J* = 9.2, 2.6 Hz, 1H), 6.26 − 6.17 (m, 1H), 5.47 − 5.34 (m, 2H), 4.54 (dd, *J* = 4.0, 2.0 Hz, 2H), 3.91 (s, 3H), 3.07 − 2.98 (m, 2H), 2.81 − 2.72 (m, 2H), 2.02 − 1.90 (m, 4H). ^13^C NMR (126 MHz, Methanol-*d*_4_) δ 157.1, 156.3, 149.1, 135.0, 133.1, 124.3, 120.2, 116.8, 115.8, 111.7, 103.7, 55.2, 48.6, 27.8, 23.7, 21.6, 20.4. HRMS: [M + H]^+^ 269.1645 (calculated for [C_17_H_21_N_2_O]^+^: 269.1654).

### 7-Phenoxy-N,N-di(prop-2-en-1-yl)-1,2,3,4-tetrahydroacridin-9-amine hydrochloride (6)

Yield 65%. mp 97.8 − 98.7 °C. Purity: 97%. ^1^H NMR (500 MHz, DMSO-*d*_6_) δ 8.40 (d, *J* = 9.2 Hz, 1H), 7.76 (dd, *J* = 9.2, 2.6 Hz, 1H), 7.53 − 7.47 (m, 2H), 7.34 (d, *J* = 2.6 Hz, 1H), 7.32 − 7.26 (m, 1H), 7.22 − 7.17 (m, 2H), 5.71 − 5.66 (m, 1H), 5.66 − 5.61 (m, 1H), 5.17 − 5.10 (m, 4H), 3.88 (d, *J* = 6.5 Hz, 4H), 3.27 (t, *J* = 6.4 Hz, 2H), 2.79 (t, *J* = 6.3 Hz, 2H), 1.92 − 1.84 (m, 2H), 1.83 − 1.75 (m, 2H). ^13^C NMR (126 MHz, DMSO-*d*_6_) δ 160.0, 156.4, 155.7, 155.3, 134.7, 134.3, 130.9, 128.0, 126.3, 125.9, 125.3, 123.0, 120.2, 119.3, 110.8, 55.0, 28.7, 26.3, 21.9, 20.7. HRMS: [M + H]^+^ 371.2114 (calculated for [C_25_H_27_N_2_O]^+^: 371.2123).

### 7-Phenoxy-N-(prop-2-en-1-yl)-1,2,3,4-tetrahydroacridin-9-amine hydrochloride (7)

Yield 23%. mp 49.6 − 50.5 °C. Purity: 95%. ^1^H NMR (500 MHz, DMSO-*d*_6_) δ 8.13 (d, *J* = 9.2 Hz, 1H), 8.01 (t, *J* = 6.4 Hz, 1H), 7.77 (d, *J* = 2.2 Hz, 1H), 7.65 (dd, *J* = 9.2, 2.3 Hz, 1H), 7.48 − 7.39 (m, 2H), 7.24 − 7.18 (m, 1H), 7.11 − 7.05 (m, 2H), 5.81 − 5.71 (m, 1H), 5.11 − 5.01 (m, 2H), 4.31 − 4.23 (m, 2H), 3.03 (t, *J* = 5.6 Hz, 2H), 2.65 (t, *J* = 5.5 Hz, 2H), 1.86 − 1.75 (m, 4H). ^13^C NMR (126 MHz, DMSO-*d*_6_) δ 156.4, 155.6, 154.1, 150.4, 135.1, 134.8, 130.8, 125.8, 124.7, 121.9, 119.6, 116.5, 116.5, 112.0, 111.7, 48.6, 28.2, 24.2, 21.8, 20.7. HRMS: [M + H]^+^ 331.1799 (calculated for [C_22_H_23_N_2_O]^+^: 331.1810).

### N,N-Di(prop-2-yn-1-yl)-1,2,3,4-tetrahydroacridin-9-amine hydrochloride (8)

Yield 17%. mp 198.1 − 199.0 °C. Purity: 95%. ^1^H NMR (500 MHz, DMSO-*d*_6_) δ 8.37 (d, *J* = 8.3 Hz, 1H), 8.17 (d, *J* = 8.4 Hz, 1H), 8.03 − 7.94 (m, 1H), 7.83 − 7.75 (m, 1H), 4.48 − 4.32 (m, 4H), 3.55 − 3.43 (m, 2H), 3.39 − 3.26 (m, 2H), 2.99 − 2.88 (m, 2H), 1.96 − 1.86 (m, 2H), 1.85 − 1.76 (m, 2H). ^13^C NMR (126 MHz, DMSO-*d*_6_) δ 159.6, 157.2, 138.2, 133.4, 128.4, 128.3, 125.8, 125.0, 120.6, 79.4, 77.2, 42.2, 28.9, 26.0, 21.8, 20.7. HRMS: [M + H]^+^ 275.1539 (calculated for [C_19_H_19_N_2_]^+^: 275.1548).

### 7-Methoxy-N,N-di(prop-2-yn-1-yl)-1,2,3,4-tetrahydroacridin-9-amine hydrochloride (12)

Yield 9%. mp 218.9 − 220.2 °C. Purity: 95%. ^1^H NMR (500 MHz, Methanol-*d*_4_) δ 8.00 (d, *J* = 9.1 Hz, 1H), 7.64 (d, *J* = 2.5 Hz, 1H), 7.62 (dd, *J* = 9.1, 2.5 Hz, 1H), 4.49 − 4.42 (m, 4H), 4.03 (s, 3H), 3.29 (t, *J* = 6.4 Hz, 2H), 3.08 (t, *J* = 6.3 Hz, 2H), 3.02 (t, *J* = 2.2 Hz, 2H), 2.08 − 2.00 (m, 2H), 1.98 − 1.91 (m, 2H). ^13^C NMR (126 MHz, Methanol-*d*_4_) δ 151.3, 151.3, 145.7, 125.7, 120.6, 118.7, 117.9, 113.2, 95.6, 70.3, 66.8, 47.7, 33.0, 20.3, 18.1, 13.6, 12.5. HRMS: [M + H]^+^ 305.1645 (calculated for [C_20_H_21_N_2_O]^+^: 305.1654).

### 7-Methoxy-N-(prop-2-yn-1-yl)-1,2,3,4-tetrahydroacridin-9-amine hydrochloride (13)

Yield 37%. mp 236.7 − 238.1 °C. Purity: 98%. ^1^H NMR (500 MHz, Metanol-*d*_4_) δ 8.00 (d, *J* = 9.1 Hz, 1H), 7.64 (d, *J* = 2.5 Hz, 1H), 7.62 (dd, *J* = 9.1, 2.5 Hz, 1H), 4.49–4.42 (m, 4H), 4.03 (s, 3H), 3.29 (t, *J* = 6.4 Hz, 2H), 3.08 (t, *J* = 6.3 Hz, 2H), 3.02 (t, *J* = 2.2 Hz, 2H), 2.08–2.00 (m, 2H), 1.98–1.91 (m, 2H). ^13^C NMR (126 MHz, Metanol-*d*_4_) δ 151.3, 151.3, 145.7, 125.7, 120.6, 118.7, 117.9, 113.2, 95.6, 70.3, 66.8, 47.7, 33.0, 20.3, 18.1, 13.6, 12.5. HRMS: [M + H]^+^ 267.1487 (calculated for [C_17_H_19_N_2_O]^+^: 267.1497).

### 7-Phenoxy-N,N-di(prop-2-yn-1-yl)-1,2,3,4-tetrahydroacridin-9-amine hydrochloride (14)

Yield 15%. mp 172.9 − 173.9 °C. Purity: 98%. ^1^H NMR (500 MHz, DMSO-*d*_6_) δ 8.42 (d, *J* = 9.2 Hz, 1H), 7.78 (dd, *J* = 9.2, 2.6 Hz, 1H), 7.52 − 7.45 (m, 3H), 7.30 − 7.24 (m, 1H), 7.22 − 7.16 (m, 2H), 4.28 − 4.15 (m, 4H), 3.34 − 3.27 (m, 4H), 2.90 (t, *J* = 6.2 Hz, 2H), 1.93 − 1.85 (m, 2H), 1.84 − 1.76 (m, 2H). ^13^C NMR (126 MHz, DMSO-*d*_6_) δ 157.8, 156.4, 155.5, 155.3, 134.6, 130.6, 128.9, 126.3, 125.9, 125.0, 122.9, 119.9, 110.2, 78.9, 76.7, 41.3, 28.5, 25.6, 21.4, 20.4. HRMS: [M + H]^+^ 367.1802 (calculated for [C_25_H_23_N_2_O]^+^: 367.1810).

### 7-Phenoxy-N-(prop-2-yn-1-yl)-1,2,3,4-tetrahydroacridin-9-amine hydrochloride (15)

Yield 26%. mp 179.1 − 180.5 °C. Purity: 96%. ^1^H NMR (500 MHz, DMSO-*d*_6_) δ 8.26 (t, *J* = 6.5 Hz, 1H), 8.19 − 8.12 (m, 2H), 7.65 (dd, *J* = 9.2, 2.4 Hz, 1H), 7.45 − 7.38 (m, 2H), 7.21 − 7.15 (m, 1H), 7.12 − 7.05 (m, 2H), 4.54 − 4.44 (m, 2H), 3.30 (t, *J* = 2.3 Hz, 1H), 3.10 − 3.02 (m, 2H), 2.78 − 2.69 (m, 2H), 1.88 − 1.75 (m, 4H). ^13^C NMR (126 MHz, DMSO-*d*_6_) δ 156.5, 154.6, 153.6, 151.0, 134.5, 130.4, 126.0, 124.1, 121.8, 118.7, 116.9, 112.5, 112.1, 80.2, 76.3, 35.9, 28.0, 24.1, 21.5, 20.3. HRMS: [M + H]^+^ 329.1645 (calculated for [C_22_H_21_N_2_O]^+^: 329.1654).

### N,N-Di(prop-2-yn-1-yl)-N'-(1,2,3,4-tetrahydroacridin-9-yl)propane-1,3-diamine dihydrochloride (20)

Yield 19%. mp 105.2 − 106.5 °C. Purity: 95%. ^1^H NMR (500 MHz, DMSO-*d*_6_) δ 8.52 (d, *J* = 8.7 Hz, 1H), 8.13 (dd, *J* = 8.6, 1.2 Hz, 1H), 8.07 (bs, 1H), 7.93 − 7.87 (m, 1H), 7.65 − 7.58 (m, 1H), 4.17 (s, 4H), 4.02 (q, *J* = 6.3 Hz, 2H), 3.94 − 3.82 (m, 2H), 3.38 − 3.22 (m, 2H), 3.09 (t, *J* = 5.7 Hz, 2H), 2.76 (t, *J* = 5.6 Hz, 2H), 2.36 − 2.20 (m, 2H), 1.95 − 1.80 (m, 4H). ^13^C NMR (126 MHz, DMSO-*d*_6_) δ 156.0, 151.4, 138.2, 132.9, 125.7, 125.4, 119.6, 116.2, 111.9, 81.9, 73.3, 49.7, 44.6, 42.0, 28.4, 25.0, 24.7, 22.0, 20.7. HRMS: [M + H]^2+^ 166.6095 (calculated for [C_22_H_27_N_3_]^2+^: 166.6097).

### N-(Prop-2-yn-1-yl)-N'-(1,2,3,4-tetrahydroacridin-9-yl)propane-1,3-diamine dihydrochloride (21)

Yield 14%. mp 151.4 − 152.3 °C. Purity: 97%. ^1^H NMR (500 MHz, DMSO-*d*_6_) δ 9.90 (bs, 2H), 8.55 (d, *J* = 8.7 Hz, 1H), 8.09 (dd, *J* = 8.6, 1.2 Hz, 1H), 8.05 (bs, 1H), 7.96 − 7.86 (m, 1H), 7.69 − 7.57 (m, 1H), 4.06 (q, *J* = 6.7 Hz, 2H), 3.98 − 3.89 (m, 2H), 3.74 (t, *J* = 2.5 Hz, 1H), 3.18 − 3.02 (m, 4H), 2.77 (t, *J* = 5.5 Hz, 2H), 2.30 − 2.13 (m, 2H), 1.95 − 1.80 (m, 4H). ^13^C NMR (126 MHz, DMSO-*d*_6_) δ 156.0, 151.3, 138.2, 133.0, 125.7, 125.5, 119.6, 116.1, 111.9, 79.9, 75.4, 44.3, 43.5, 35.8, 28.4, 26.7, 24.7, 22.0, 20.7. HRMS: [M + H]^2+^ 147.6016 (calculated for [C_19_H_25_N_3_]^2+^: 147.6019).

### N'-(6-Chloro-1,2,3,4-tetrahydroacridin-9-yl)-N,N-di(prop-2-yn-1-yl)propane-1,3-diamine dihydrochloride (22)

Yield 11%. mp 182.2 − 183.5 °C. Purity: 98%. ^1^H NMR (500 MHz, DMSO-*d*_6_) δ 8.48 (d, *J* = 9.3 Hz, 1H), 8.18 (d, *J* = 2.3 Hz, 1H), 8.15 (bs, 1H), 7.56 (dd, *J* = 9.2, 2.2 Hz, 1H), 4.11 (s, 4H), 3.96 (q, *J* = 6.5 Hz, 2H), 3.85 − 3.78 (m, 2H), 3.30 − 3.19 (m, 2H), 3.02 (t, *J* = 5.1 Hz, 2H), 2.67 (t, *J* = 5.2 Hz, 2H), 2.29 − 2.15 (m, 2H), 1.88 − 1.75 (m, 4H). ^13^C NMR (126 MHz, DMSO-*d*_6_) δ 155.85, 151.86, 139.0, 137.3, 128.0, 125.8, 118.3, 114.7, 112.3, 81.9, 73.3, 49.6, 44.6, 42.0, 28.4, 24.8, 24.5, 21.8, 20.5. HRMS: [M + H]^2+^ 183.5901 (calculated for [C_22_H_26_ClN_3_]^2+^: 183.5902).

### N-(6-Chloro-1,2,3,4-tetrahydroacridin-9-yl)-N'-(prop-2-yn-1-yl)propane-1,3-diamine dihydrochloride (23)

Yield 25%. mp 184.5 − 185.2 °C. Purity: 97%. ^1^H NMR (500 MHz, DMSO-*d*_6_) δ 9.86 (bs, 2H), 8.49 (d, *J* = 9.3 Hz, 1H), 8.19 − 8.05 (m, 2H), 7.55 (dd, *J* = 9.2, 2.3 Hz, 1H), 3.98 (q, *J* = 6.7 Hz, 2H), 3.87 (s, 2H), 3.68 (t, *J* = 2.5 Hz, 1H), 3.12 − 2.91 (m, 4H), 2.68 (t, *J* = 5.0 Hz, 2H), 2.23 − 2.08 (m, 2H), 1.91 − 1.72 (m, 4H). ^13^C NMR (126 MHz, DMSO-*d*_6_) δ 155.7, 151.7, 138.9, 137.3, 128.1, 125.8, 118.3, 114.6, 112.3, 79.8, 75.4, 44.3, 43.4, 35.8, 28.4, 26.4, 24.6, 21.8, 20.6. HRMS: [M + H]^2+^ 164.5822 (calculated for [C_19_H_24_ClN_3_]^2+^: 164.5824).

### N-(7-Methoxy-1,2,3,4-tetrahydroacridin-9-yl)-N',N'-di(prop-2-yn-1-yl)-propane-1,3-diamine dihydrochloride (24)

Yield 54%. mp 171.6 − 173.0 °C. Purity: 96%. ^1^H NMR (500 MHz, DMSO-*d*_6_) δ 7.99 (bs, 1H), 7.97 (d, *J* = 9.2 Hz, 1H), 7.79 (d, *J* = 2.3 Hz, 1H), 7.50 (dd, *J* = 9.2, 2.4 Hz, 1H), 4.15 − 4.00 (m, 4H), 3.95 (s, 3H), 3.93 − 3.88 (m, 2H), 3.79 − 3.71 (m, 2H), 3.23 − 3.14 (m, 2H), 3.02 (t, *J* = 5.6 Hz, 2H), 2.75 (t, *J* = 5.5 Hz, 2H), 2.22 − 2.12 (m, 2H), 1.86 − 1.75 (m, 4H). ^13^C NMR (126 MHz, DMSO-*d*_6_) δ 156.8, 154.8, 150.0, 132.6, 124.1, 120.9, 117.6, 111.5, 103.6, 81.1, 73.4, 56.4, 49.4, 43.8, 41.8, 28.0, 25.3, 24.9, 21.9, 20.4. HRMS: [M + H]^2+^ 181.6148 (calculated for [C_23_H_29_N_3_O]^2+^: 181.6150).

### N-(7-Phenoxy-1,2,3,4-tetrahydroacridin-9-yl)-N',N'-di(prop-2-yn-1-yl)-propane-1,3-diamine dihydrochloride (25)

Yield 36%. mp 176.3 − 177.7 °C. Purity: 96%. ^1 1^H NMR (500 MHz, DMSO-*d*_6_) δ 8.15 (d, *J* = 9.2 Hz, 1H), 8.00 − 7.94 (m, 1H), 7.90 (bs, 1H), 7.62 (dd, *J* = 9.2, 2.3 Hz, 1H), 7.47 − 7.39 (m, 2H), 7.23 − 7.16 (m, 1H), 7.09 (d, *J* = 8.2 Hz, 2H), 4.00 (s, 4H), 3.81 (q, *J* = 6.0 Hz, 2H), 3.77 − 3.70 (m, 2H), 3.16 − 3.07 (m, 2H), 3.07 − 2.99 (m, 2H), 2.77 − 2.66 (m, 2H), 2.09 − 2.01 (m, 2H), 1.87 − 1.77 (m, 4H). ^13^C NMR (126 MHz, DMSO-*d*_6_) δ 156.5, 155.0, 153.5, 150.9, 134.4, 130.4, 125.6, 124.1, 121.7, 118.7, 117.2, 112.3, 111.7, 81.0, 73.6, 49.4, 44.1, 41.7, 28.1, 24.9, 24.6, 21.7, 20.3. HRMS: [M + H]^2+^ 212.6225 (calculated for [C_28_H_31_N_3_O]^2+^: 212.6228).

### N-(7-Phenoxy-1,2,3,4-tetrahydroacridin-9-yl)-N'-(prop-2-yn-1-yl)-propane-1,3-diamine dihydrochloride (26)

Yield 16%. mp 178.6 − 180.0 °C. Purity: 96%. ^1^H NMR (500 MHz, DMSO-*d*_6_) δ 9.75 (bs, 2H), 8.10 (d, *J* = 9.2 Hz, 1H), 7.99 (d, *J* = 2.2 Hz, 1H), 7.87 (t, *J* = 5.8 Hz, 1H), 7.61 (dd, *J* = 9.2, 2.3 Hz, 1H), 7.47 − 7.40 (m, 2H), 7.22 − 7.17 (m, 1H), 7.11 − 7.06 (m, 2H), 3.88 − 3.84 (m, 2H), 3.84 − 3.80 (m, 2H), 3.67 (t, *J* = 2.5 Hz, 1H), 3.08 − 3.01 (m, 2H), 2.95 (t, *J* = 7.0 Hz, 2H), 2.76 − 2.68 (m, 2H), 2.05 − 1.94 (m, 2H), 1.86 − 1.76 (m, 4H). ^13^C NMR (126 MHz, DMSO-*d*_6_) δ 156.5, 155.0, 153.6, 150.8, 134.3, 130.4, 125.6, 124.2, 121.7, 118.8, 117.1, 112.2, 111.8, 79.5, 75.0, 43.6, 43.1, 35.5, 28.1, 26.3, 24.7, 21.7, 20.3. HRMS: [M + H]^2+^ 193.6148 (calculated for [C_25_H_29_N_3_O]^2+^: 193.6150).

### 7-Chloro-N-(prop-2-en-1-yl)quinolin-4-amine hydrochloride (27)

Yield 90%. mp 179.4 − 181.2 °C. Purity: 99%. ^1^H NMR (500 MHz, DMSO-*d*_6_) δ 10.00 (t, *J* = 5.7 Hz, 1H), 8.74 (d, *J* = 9.1 Hz, 1H), 8.53 (d, *J* = 7.0 Hz, 1H), 8.13 (d, *J* = 2.1 Hz, 1H), 7.74 (dd, *J* = 9.1, 2.1 Hz, 1H), 6.74 (d, *J* = 7.1 Hz, 1H), 5.98 − 5.89 (m, 1H), 5.29 − 5.18 (m, 2H), 4.23 − 4.16 (m, 2H). ^13^C NMR (126 MHz, DMSO-*d*_6_) δ 155.9, 143.0, 139.0, 138.3, 132.7, 127.3, 126.3, 119.4, 117.4, 115.9, 99.4, 45.4. HRMS: [M + H]^+^ 219.0680 (calculated for [C_12_H_12_ClN_2_]^+^: 219.0684).

### 7-Chloro-N-(prop-2-yn-1-yl)quinolin-4-amine hydrochloride (28)

Yield 50%. mp 233.6 − 235.0 °C. Purity: 99%. ^1^H NMR (500 MHz, DMSO-*d*_6_) δ 10.22 (bs, 1H), 8.68 (dd, *J* = 13.2, 7.6 Hz, 2H), 8.15 (d, *J* = 2.1 Hz, 1H), 7.83 − 7.70 (m, 1H), 6.89 (d, *J* = 7.5 Hz, 1H), 4.46 − 4.34 (m, 2H), 3.40 (t, *J* = 2.4 Hz, 1H). ^13^C NMR (126 MHz, DMSO-*d*_6_) δ 155.5, 143.5, 138.9, 138.5, 127.6, 126.2, 119.5, 116.0, 99.7, 78.8, 75.7, 32.6. HRMS: [M + H]^+^ 217.0524 (calculated for [C_12_H_10_ClN_2_]^+^: 217.0527).

### Evaluation of the inhibitory activity towards human AChE and BChE

The catalytic activity of both cholinesterases was determined by standard Ellman’s method adapted for 96-well plates^35^. All tested inhibitors were freshly prepared in 50% DMSO/50% methanol at 10 mM concentration as stock solutions and then diluted in 0.1 M phosphate buffer, pH 7.4. The reaction mixture contained *h*AChE (70 ng/mL protein final concentration) or *h*BChE (220 ng/mL protein); studied inhibitor at required concentration (0.2–100 μM) and 500 μM 5,5′-dithiobis-2-nitrobenzoic acid (DTNB) in 20 mM sodium phosphate buffer (pH 7.4). The mixture was pre-incubated at 37 °C for 15 min and subsequently substrate (acetylthiocholine iodide or butyrylthiocholine iodide) was added to the final concentration of 1000 μM. The final volume of the reaction was 100 μL. The catalytic activity was evaluated as the amount of product (%) formed by enzyme after 10 min of incubation at 37 °C. The IC_50_ values from three independent experiments for each inhibitor concentration in triplicate were calculated using non-linear regression curve analysis in Prism version 7 Software (GraphPad Software Inc., San Diego, CA).

### Kinetic study of AChE inhibition

Compound with the highest inhibition potential against *h*AChE was further analysed regarding its inhibition kinetics parameters (inhibition mechanism and inhibitory constant). Thus, esterase activity assay was carried out at various concentrations of substrate ATChI (ranging from 25 to 2000 µM) and various concentrations of tested compound (0.01, 0.02, and 0.05 µM). Inhibition mechanism and kinetic constant were determined by non-linear regression and double reciprocal method by Lineweaver-Burk using GraphPad Prism version 7^51^.

### Determination of the inhibitory potential towards human MAO-A and MAO-B

The *h*MAO-A and *h*MAO-B enzymes were purchased from Sigma-Aldrich (St. Louis, MO, USA). The reaction mixture contained *h*MAO-A (2.5 µg/mL protein final concentration) or *h*MAO-B (6.25 µg/mL protein final concentration) enzyme and tested compound in final concentration of 1 and 10 µM in 50 mM potassium phosphate buffer with 20% (*v*/*v*) glycerol (pH 7.5). The mixture was pre-incubated at 37 °C for 5 min and subsequently substrate kynuramine was added to the final concentration of 60 µM in the case of *h*MAO-A and 30 µM in the case of *h*MAO-B. The final volume of reaction mixture was 0.1 mL. The whole reaction mixture was incubated at 37 °C for 30 min. The reaction was stopped by the addition of 200 µL acetonitrile/methanol mixture (ratio 1:1) and cooling down to 0 °C. The sample was then centrifuged (16.500× g) for 10 min. The deamination product of kynuramine formed during the enzymatic reaction 4-hydroxyquinoline (4-HQ) was determined by HPLC–MS on a 2.1 mm × 50 mm, 1.8 µm Zorbax RRHD Eclipse plus C18 column (Agilent) by using a 6470 Series Triple Quadrupole mass spectrometer (Agilent) (electrospray ionisation – positive ion mode). Three MRM transitions were followed for kynuramine (165.1 = > 30.2, 165.1 = > 118.0, 165.1 = > 136.0) and 4-HQ (146.1 = > 51.1, 146.1 = > 77.0, 146.1 = > 91.0). Eluents: (A) 0.1% formic acid in water; (B) 0.1% formic acid in acetonitrile.

### Computational chemistry studies

The X-ray models (*h*AChE - PDB IDs: 4EY7, 4M07, 7RB6; *h*MAO-B - PDB IDs: 2V5Z, 3PO7, 4CRT) were downloaded from rcsb.org and prepared for IFD in Protein Preparation Wizard in Schrodinger 2021–4. The enzyme preparation involved the separation of a single protein chain, removing water and inorganic molecules, adding hydrogens, protonation corresponding to pH = 7.0 ± 2.0, reconstruction of H-bond networks, checking steric atom clashes and distorted bonds, and geometrical minimisation up to RMSD of 0.3 Å relative to the starting geometry. In the cases of 4EY7, 4M07, 7RB6, the missing residues were added with CrossLink utility of Schrodinger 2021–4 and homologically modelled in an implicit water model. In the 4CRT model, it was necessary to manually split the ligand ASS234 propargyl group connected to the nitrogen atom of the flavin moiety in FAD and cap the ligand propargyl group with a hydrogen atom in its distal end. All the enzyme models, along with the co-crystalised ligands and co-factors, were properly checked, repaired, parameterised, and optimised using OPLS_2005 force field.

The compounds **15** and **23** were created in HyperChem 8.0 and geometrically optimised with semi-empirical QM method PM3. Next, the ligands were reparametrised with the force field OPLS_2005 and polarised for pH = 7.0 ± 2.0 in LigPrep utility of Schrodinger 2021–4. For both ligands, up 32 locally optimised conformers and protomers were automatically generated. In the same way, the co-crystalised ligands were prepared for IFD in LigPrep utility to evaluate if the calculation protocol can reproduce the geometrical structure of the ligand-enzyme complexes determined by X-ray. IFD was performed on molecular mechanics level with OPLS_2005 force field in Schrodinger 2021–4, which combines Glide version 9.3 and Prime version 6.6 programs to find the optimum ligand position in the enzyme. For better management of the *in silico* studies, a simple bash script was developed to distribute the IFD calculation tasks in a Linux-based supercomputer. The cubic gridbox with the edge of 30 Å was centred on the co-crystalised ligands, allowing all residues involved in it to change their geometry. The IFD protocol was set to perform the calculations with extended precision (XP), which enabled to test with additional sampling up to 80 conformers for each ligand. Since the IFD algorithm performs the calculation in a deterministic way, molecular docking was not repeated because restarting the task from the same starting conditions provides the same results. For each IFD task, 8 CPUs were employed in parallel.

Hybridised QM/MM recalculations of the binding modes obtained by IFD were performed using qsite_binding_energies.py Python script, which employs QSite & Jaguar 11.4 of Schrodinger 2021–4. The script was set to treat the ligands, the FAD co-factor, residues Asp74, Trp86, Trp286, and His447 in *h*AChE and residues Leu171, Ile198, Ile199, and Tyr326 in *h*MAO-B quantum mechanically, while the rest of the protein chain was simulated with a molecular mechanistic method and OPLS_2005 force field. In the IFD outputs using 2V5Z and 3PO7 enzyme models, it was necessary to disconnect manually the flavin moiety of FAD from Cys397 and to cap free valences with hydrogen atoms. The QM/MM protocol started with a local pre-minimisation and continued with splitting the ligand-enzyme complex into separated parts. In this way, the gas phase potential energies of the ligand (E(L)), the enzyme (E(E)) and the ligand-enzyme complex (E(C)) were calculated in parallel, giving the binding energy estimate as ΔE = E(C) - E(L) - E(E). The QM region was simulated with M06-2X/CC-PVTZ(-F)++ method using residue hydrogen caping for interfacing the QM and MM parts. The MM region was approximated with OPLS_2005 force field. The QM/MM tasks were performed in parallel using 128 CPUs and 250 GB RAM. Thanks to the application of a considerably large basis set in the QM calculations, the basis set superimposition error (i.e. BSSE) was neglected in this study. Comparison of the QM/MM predicted conformations of the co-crystalised ligands with the original poses in the selected enzyme X-ray models resulted in RMSD values below 3 Å.

The reaction coordinate for covalent binding of **15** to the *sp^2^* flavine nitrogen atom in the FAD co-factor in *h*MAO-B was predicted from the QM/MM refined binding modes provided by IFD. The calculation consisted in a stepwise distance contraction between the terminal *sp* carbon atom of the *N-*propargyl moiety of **15** and the *sp^2^* nitrogen atom (i.e. N5) in the FAD co-factor in *h*MAO-B. The relaxed scanning of the reaction coordinate was performed in all three *h*MAO-B models (PDB ID: 2V57, 3PO7, 4CRT), over 19 regular steps, and ended when the scanned distance reached 1.0 Å. The protein molecules of *h*MAO-B were simulated by MM with OPLS_2005 force field whereas the ligand **15** and the FAD co-factor were calculated by QM with DFT B3LYP/LAV3P++** method. This QM/MM relaxed scan utilised 128 CPUs in parallel and 250 GB RAM. All analyses and graphical visualisation were performed with tools available in Schrodinger 2021–4.

Supplementary IFD studies were performed for compounds **1 − 28**, THA, 6-chlolorotacrine, 7-MEOTA, 7-PhOTA, clorgyline, and pargyline in the same way as mentioned above. The ligand molecules were built up in HyperChem 8.0, geometrically optimised, reparametrised by OPLS 2005 force field in Schrodinger 2021–4 and polarised for pH = 7.0 ± 2.0. Based on the optimal resolutions and R-free factors, X-ray protein models of *h*BChE (PDB ID: 6QAC) and *h*MAO-A (PDB ID: 2Z5X) were selected, downloaded from rcsb.org database, and prepared for IFD in Protein Preparation Wizard in Schrodinger 2021–4. Using the *h*AChE (PDB ID: 4M07) and *h*MAO-B (PDB ID: 3PO7) models from the previous *in silico* stage, all the studied compounds were evaluated by IFD in Schrodinger 2021–4 for interactions in binding sites of *h*AChE, *h*BChE, *h*MAO-A, and *h*MAO-A. To achieve the best performance in all IFD calculations, the petascale supercomputer Karolina was employed.

### In vitro BBB permeation assay

The PAMPA was used as the non-cell-based *in vitro* assay to predict BBB penetration carried out in a coated 96-well membrane filter^44^^,^^52^. The filter membrane of the donor plate was coated with polar brain lipid (PBL, Avanti, USA) in dodecane (4 µL of 20 mg/mL PBL in dodecane) and the acceptor well was filled with 300 µL of PBS buffer (pH 7.4; V_A_). Tested compounds were dissolved first in DMSO and then diluted with PBS (pH 7.4) to achieve the final concentration of 100 µM in the donor well. The concentration of DMSO did not exceed 0.5% (v/v) in the donor solution. An aliquot of 300 µL of the donor solution (V_D_) was added to the donor wells and the donor filter plate was carefully put on the acceptor plate so that coated membrane was "in touch" with both donor solution and acceptor buffer. The test compound diffused from the donor well through the polar brain lipid membrane (Area = 0.28 cm^2^) to the acceptor well. The concentration of the tested compound in both the donor and acceptor wells was assessed after 3, 4, 5, and 6 h of incubation, respectively, in quadruplicate using a multi-plate reader Spark (Tecan GmbH, Grödig, Austria) at the maximum absorption wavelength of each compound. Also prepared were solutions at the theoretical equilibrium of the given compound (i.e. the theoretical concentration if the donor and acceptor compartment were simply combined). Concentration of the compounds in the donor and acceptor well and the equilibrium concentration were calculated from the standard curve and expressed as the permeability (*Pe*) according the Equation [1]:

$$Pe=C\times -ln(1-\frac{{\left[\mathrm{drug}\right]}_{\mathrm{acceptor}}}{{\left[\mathrm{drug}\right]}_{\mathrm{equilibrium}}}),$$

where

$$C=(\frac{V_{D}\times V_{A}}{(V_{D}+V_{A})\times \mathrm{Area}\times \mathrm{Time}}).$$

### Cytotoxicity evaluation

The cytotoxicity of tested compounds was assessed on liver hepatocellular carcinoma (HepG2, ATCC, Mannassas, VA) and neuroblastoma (SH-SY5Y, ATCC) cell line using the MTT (Sigma-Aldrich, St. Louis, MO) reduction assay that has been slightly modified^43^^,^^53^.

Briefly, HepG2 and SH-SY5Y cells were seeded into 96-well plates in 100 µL and density of 15 × 10^3^ and 20 × 10^3^ cells per well, respectively. Cells were allowed to attach overnight. The stock solutions of tested compounds were prepared in DMSO (Sigma-Aldrich), further serially diluted in cultivation medium and added to the cells in 96-well culture plate. The final concentration of DMSO was less than 0.25% per well. After 24 h was the medium aspirated and 100 µL MTT solution (0.5 mg/mL) in serum free DMEM medium was added to each well. The cells were then incubated for one hour. The medium was then aspirated and purple crystals of MTT formazan were dissolved in 100 µL DMSO under shaking. The absorbance was measured with a multimode microplate reader Spark^®^ (Tecan Trading AG, Männedorf, Switzerland) at test wavelength of 570 nm.

The IC_50_ values were calculated using four parametric non-linear regression by statistic GraphPad Prism software version 5.04 (GraphPad Software Inc., San Diego, CA) from the logarithmic dose–response curve. The data were obtained from three independent experiments performed in triplicates. The IC_50_ values were expressed as a mean ± standard error of the mean (SEM).

## Supplementary Material

Supplemental Material
