# Supplementary material for: Privileged multi-target directed propargyl-tacrines combining cholinesterase and monoamine oxidase inhibition activities
Source: J Enzyme Inhib Med Chem. 2022 Sep 21;37(1):2605–20. doi: 10.1080/14756366.2022.2122054 (PMC9518283; doi:10.1080/14756366.2022.2122054)
Supplement: Supplemental Material [file IENZ_A_2122054_SM7212.pdf]

Privileged Multi-Target Directed Propargyl-Tacrines Combining Cholinesterase and Monoamine Oxidase Inhibition Activities

SUPPORTING INFORMATION

Zofia Chrienova[a,#], Eugenie Nepovimova[a,#], Rudolf Andrys[a], Rafael Dolezal[b], Jana Janockova[b], Lubica Muckova[b,c], Lenka Fabova[d], Ondrej Soukup[b], Patrik Oleksak[a], Martin Valis[b,e], Jan Korabecny[b,c,*], Kamil Kuca[a,*] and José Marco-Contelles[f,*]

[a] *Department of Chemistry, Faculty of Science, University of Hradec Kralove, Rokitanskeho 62, 500 03 Hradec Kralove, Czech Republic.*

[b] *Biomedical Research Centre and Department of Neurology, University Hospital Hradec Kralove, Sokolska 581, 500 05 Hradec Kralove, Czech Republic.*

[c] *Department of Toxicology and Military Pharmacy, Faculty of Military Health Sciences, University of Defence, Trebesska 1575, 500 01 Hradec Kralove, Czech Republic.*

[d] *Department of Pharmaceutical Chemistry and Pharmaceutical Analysis, Faculty of Pharmacy in Hradec Kralove, Charles University in Prague, Heyrovskeho 1203, 500 05 Hradec Kralove, Czech Republic.*

[e] *Faculty of Medicine in Hradec Kralove, Charles University in Prague, Simkova 870/13, 500 03 Hradec Kralove, Czech Republic.*

[f] *Laboratory of Medicinal Chemistry, Institute of General Organic Chemistry (CSIC), Juan de La Cierva 3, 28006-Madrid, Spain.*

[#] *Z.C. and E.N. contributed equally.*

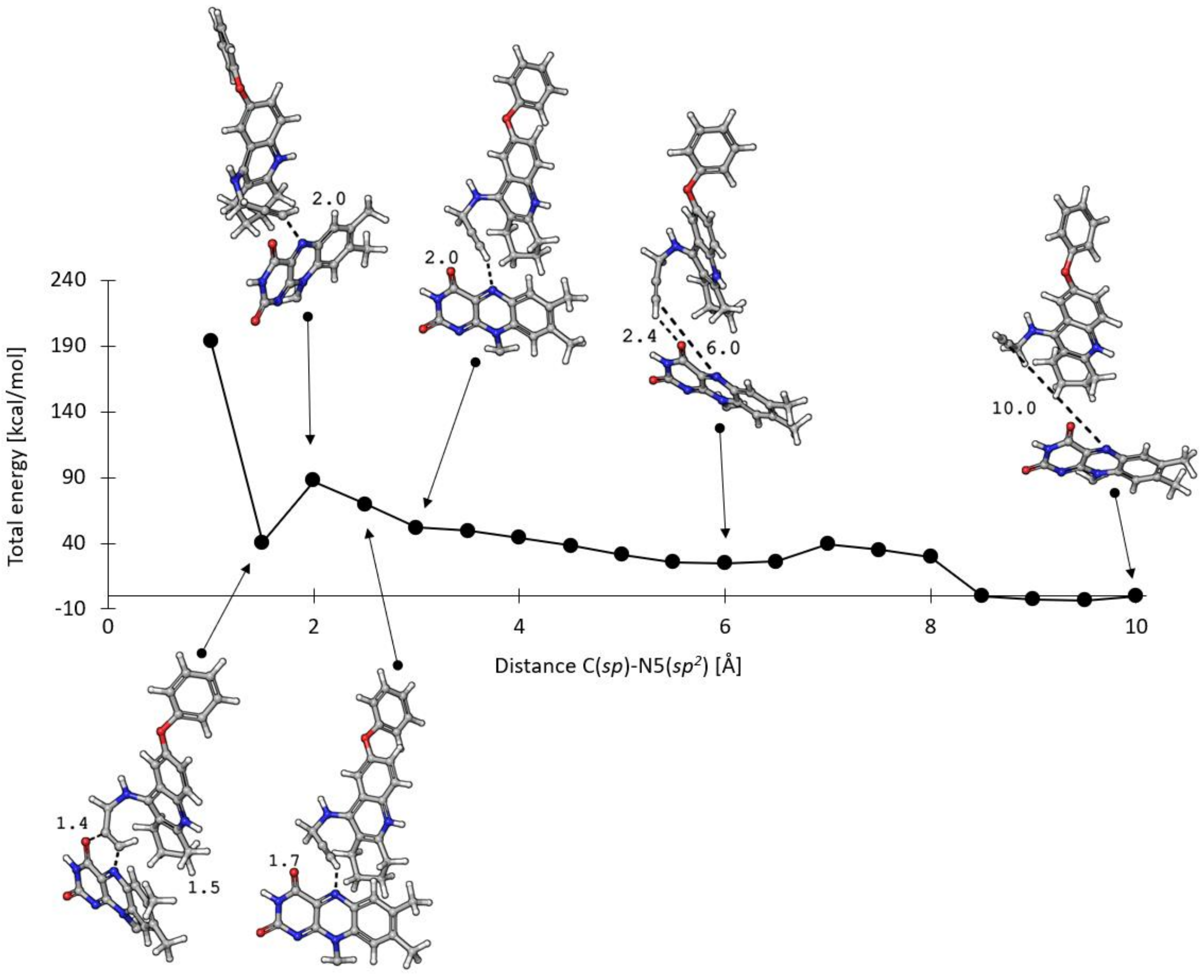

Figure S1. Simulated reaction coordinate for covalent binding of **15** to N5 atom of the FAD co-factor within *h*MAO-B (PDB ID: 2V5Z), consisting of 19 steps. The QM/MM simulation was performed by DFT B3LYP/LAV3P++**/OPLS_2005 method, utilizing the relaxed scan technique available in QSite & Jaguar 11.4 of Schrodinger 2021-4. The QM region involved the ligand **15** and the FAD co-factor. The protein 2V5Z was approximated with OPLS_2005 force field and its structure is not displayed for the sake of clarity. The displayed distances are given in Å.

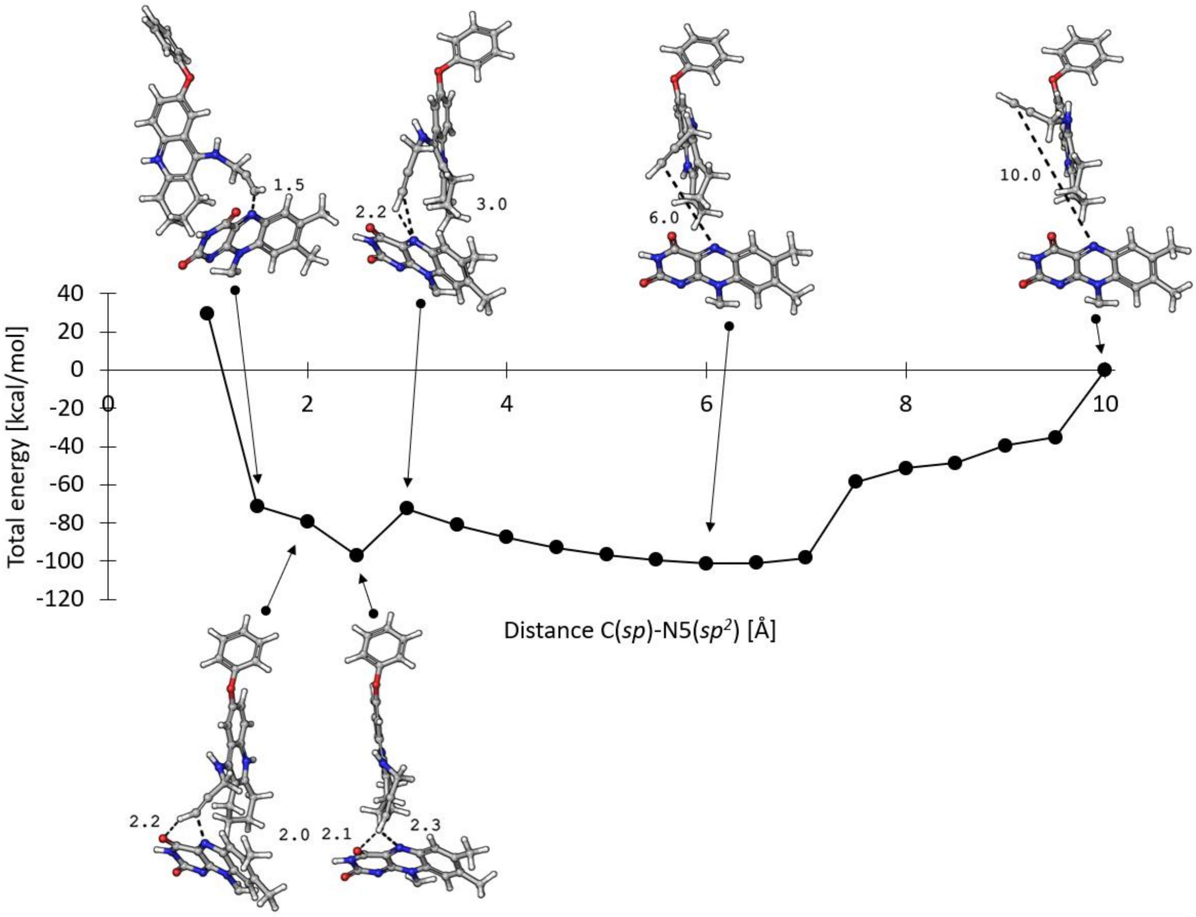

Figure S2. Simulated reaction coordinate for covalent binding of **15** to N5 atom of the FAD co-factor within *h*MAO-B (PDB ID: 4CRT), consisting of 19 steps. The QM/MM simulation was performed by DFT B3LYP/LAV3P++**/OPLS_2005 method, utilizing the relaxed scan technique available in QSite & Jaguar 11.4 of Schrodinger 2021-4. The QM region involved the ligand **15** and the FAD co-factor. The protein 4CRT was approximated with OPLS_2005 force field and its structure is not displayed for the sake of clarity. The displayed distances are given in Å.

Table S1. Predicted binding energies of **23** in three models of *h*AChE by IFD and QM/MM (DFT M06-2X/CC-PVTZ(-F)++/OPLS_2005) calculations in Schrodinger 2021-4.

***h*AChE model PDB ID**	**IFD score [kcal/mol]**	**Glide score [kcal/mol]**	**ΔE(QM/MM) [kcal/mol]**
4EY7	-22895.6	-15.0	-408.4
4M0E	-22838.4	-18.5	-491.2
7RB6	-23715.0	-15.8	-474.1

Table S2. Predicted binding energies of **15** in three models of *h*MAO-B by IFD and QM/MM (DFT M06-2X/CC-PVTZ(-F)++/OPLS_2005) calculations in Schrodinger 2021-4.

***h*MAO-B model PDB ID**	**IFD score [kcal/mol]**	**Glide score [kcal/mol]**	**QM/MM [kcal/mol]**
2V5Z	-22236.5	-13.9	-71.1
3PO7	-22222.1	-13.5	-125.9
4CRT	-22343.2	-12.8	-47.7

Table S3. Predicted binding energies of the studied compounds in *h*AChE (PDB ID: 4M0E), *h*BChE (PDB ID: 6QAC), *h*MAO-A (PDB ID: 2Z5X), and *h*MAO-B (PDB ID: 3PO7) by IFD with OPLS_2005 force field in Schrodinger 2021-4. Relatively strong interaction energies and scores are highlighted in red, weak interactions are colored in blue.

Cmp.	IFD score [kcal/mol]				Glide score [kcal/mol]			
	*h*AChE	*h*BChE	*h*MAO-A	*h*MAO-B	*h*AChE	*h*BChE	*h*MAO-A	*h*MAO-B
**1**	-22728.9	-23212.3	-22846.2	-22194.3	-14.6	-9.4	-10.4	-11.5
**2**	-22771.8	-23219.8	-22860.2	-22149.6	-14.3	-8.9	-11.3	-10.9
**3**	-22786.9	-23256.4	-22869.5	-22219.8	-15.4	-9.3	-13.6	-11.7
**4**	-22753.7	-23268.7	-22836.9	-22207.0	-11.2	-10.6	-10.3	-10.1
**5**	-22819.4	-23268.2	-22809.4	-22218.6	-13.4	-9.3	-9.9	-10.6
**6**	-22737.4	-23303.6	-22881.7	-22234.0	-14.6	-12.0	-12.7	-11.4
**7**	-22748.8	-23288.9	-22909.8	-22203.6	-13.7	-13.1	-14.4	-12.4
**8**	-22708.5	-23244.7	-22829.4	-22181.5	-15.5	-8.7	-10.8	-11.8
**9**	-22715.0	-23254.4	-22831.9	-22203.0	-13.5	-8.6	-11.5	-13.0
**10**	-22744.8	-23280.8	-22834.9	-22210.8	-11.9	-10.2	-10.7	-11.6
**11**	-22744.7	-23264.9	-22870.8	-22209.6	-14.9	-9.6	-13.4	-12.3
**12**	-22739.5	-23278.5	-22873.4	-22179.4	-12.2	-9.0	-10.2	-10.7
**13**	-22810.5	-23280.5	-22821.8	-22174.5	-14.6	-8.5	-10.3	-9.4
**14**	-22747.2	-23299.9	-22935.8	-22220.6	-12.3	-12.5	-12.3	-10.5
**15**	-22749.1	-23275.0	-22911.4	-22222.1	-14.1	-9.8	-12.7	-13.5
**20**	-22745.4	-23260.2	-22874.1	-22215.0	-14.4	-10.4	-12.1	-13.9
**21**	-22762.7	-23275.6	-22860.9	-22177.3	-15.1	-11.0	-11.4	-11.6
**22**	-22784.5	-23295.7	-22941.2	-22252.1	-13.7	-11.4	-14.2	-14.0
**23**	-22838.4	-23300.6	-22899.4	-22251.5	-18.5	-10.5	-10.8	-12.9
**24**	-22769.0	-23297.0	-22905.8	-22222.8	-13.6	-9.8	-12.1	-13.6
**25**	-22791.6	-23364.8	-22909.0	-22298.1	-12.9	-14.8	-13.7	-14.7
**26**	-22863.9	-23341.2	-22901.1	-22243.8	-18.6	-13.3	-10.6	-12.6
**27**	-22778.0	-23254.0	-22835.5	-22188.6	-14.1	-9.7	-10.0	-9.5
**28**	-22787.8	-23297.6	-22812.6	-22171.6	-12.8	-8.2	-8.5	-8.7
**THA**	-22727.6	-23290.6	-22812.3	-22136.3	-12.9	-9.1	-10.8	-9.2
**6-Cl-THA**	-22721.0	-23241.3	-22817.5	-22165.8	-13.7	-9.2	-9.1	-9.4
**7-MEOTA**	-22750.0	-23248.4	-22828.7	-22175.6	-11.5	-9.5	-10.9	-9.4
**7-PhOTA**	-22712.0	-23260.2	-22872.9	-22168.2	-13.4	-10.5	-12.8	-11.5
**clorgyline**	-22662.3	-23219.3	-22816.2	-22109.7	-8.5	-8.4	-9.1	-9.2
**pargyline**	-22587.7	-23198.3	-22775.5	-22099.3	-7.1	-7.1	-7.3	-8.1

Table S4. Predicted top scoring binding modes of the studied compounds in *h*AChE (PDB ID: 4M0E) by IFD with OPLS_2005 force field in Schrodinger 2021-4.

**1**-*h*AChE	**2**-*h*AChE	**3**-*h*AChE	**4**-*h*AChE
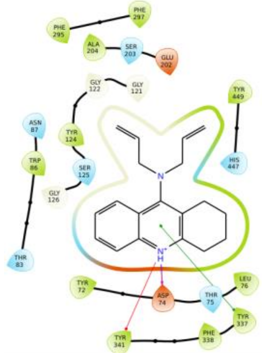	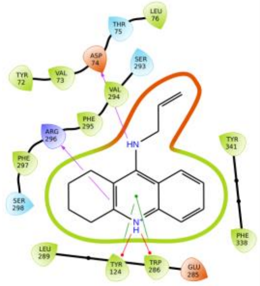	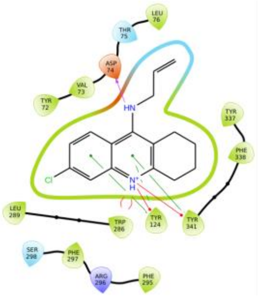	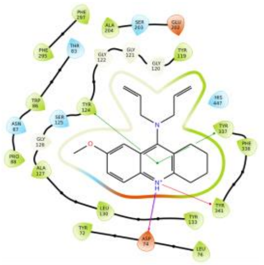
**5**-*h*AChE	**6**-*h*AChE	**7**-*h*AChE	**8**-*h*AChE
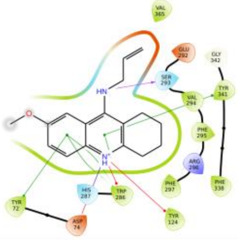	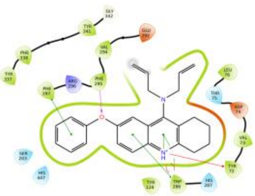	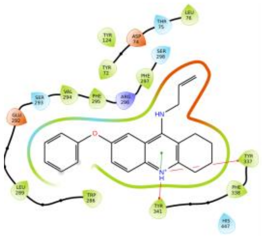	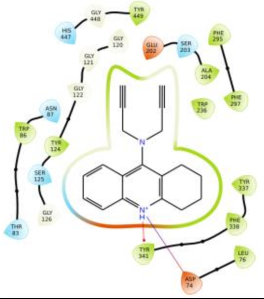
**9**-*h*AChE	**10**-*h*AChE	**11**-*h*AChE	**12**-*h*AChE
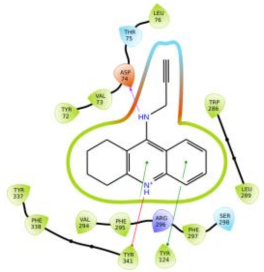	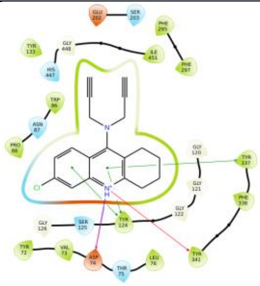	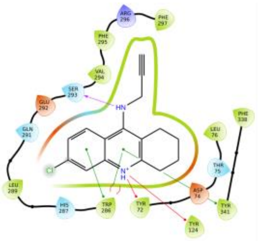	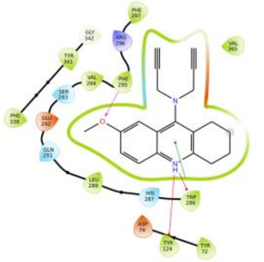
**13**-*h*AChE	**14**-*h*AChE	**15**-*h*AChE	**20**-*h*AChE
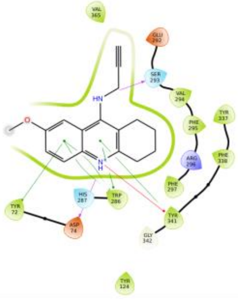	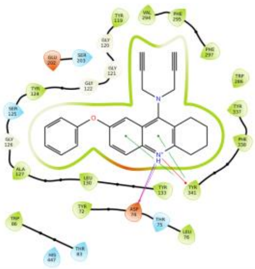	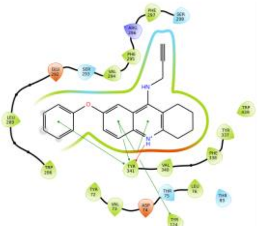	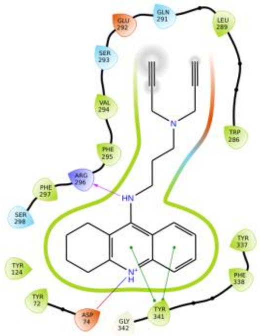
**21**-*h*AChE	**22**-*h*AChE	**23**-*h*AChE	**24**-*h*AChE

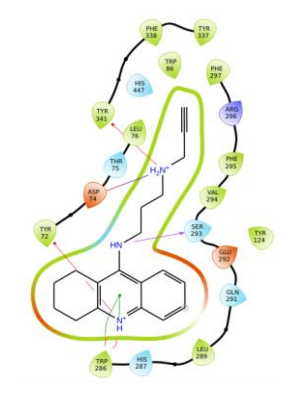	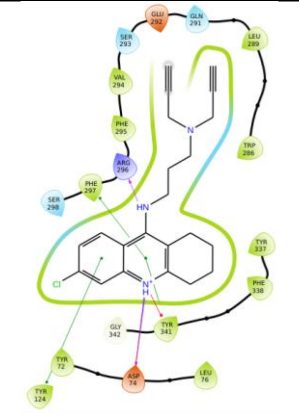	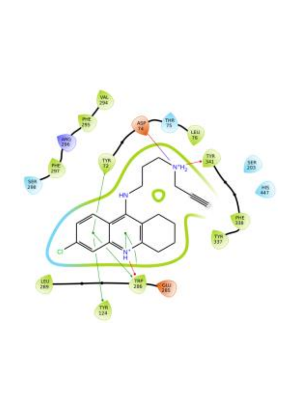	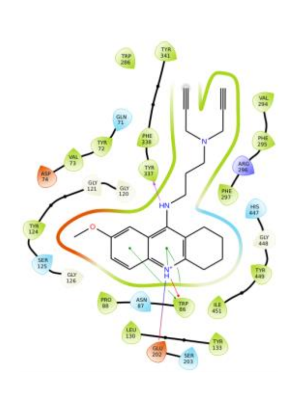
**25**-*h*AChE	**26**-*h*AChE	**27**-*h*AChE	**28**-*h*AChE
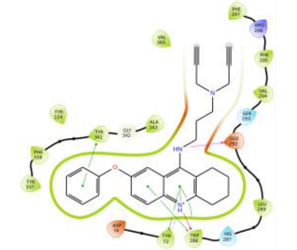	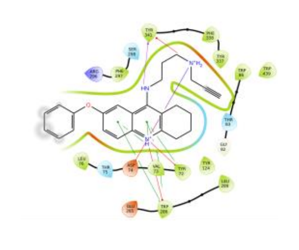	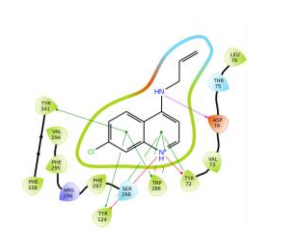	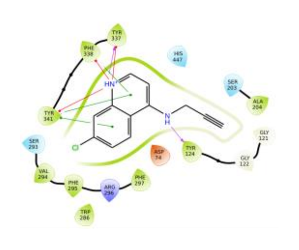
**THA**-*h*AChE	**6-Cl-THA**-*h*AChE	**7-MEOTA**-*h*AChE	**7-PhOTHA**-*h*AChE
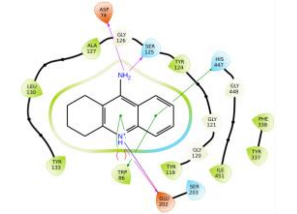	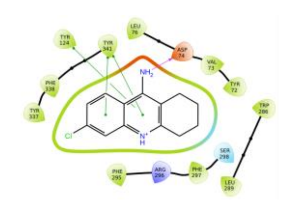	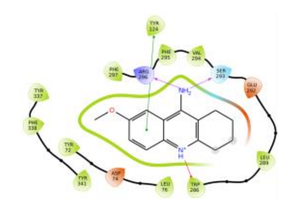	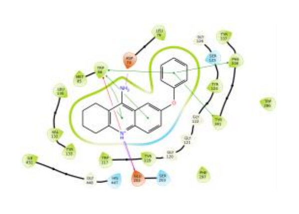
**clorgyline**-*h*AChE	**pargyline**-*h*AChE		
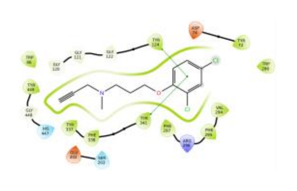	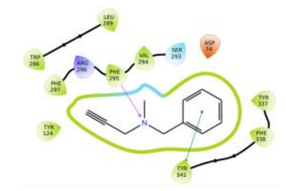		

Table S5. Predicted top scoring binding modes of the studied compounds in *h*BChE (PDB ID: 6QAC) by IFD with OPLS_2005 force field in Schrodinger 2021-4.

**1**-*h*BChE	**2**-*h*BChE	**3**-*h*BChE	**4**-*h*BChE
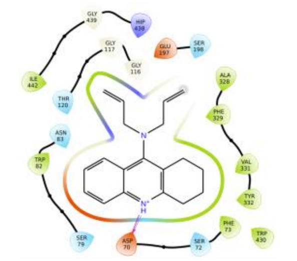	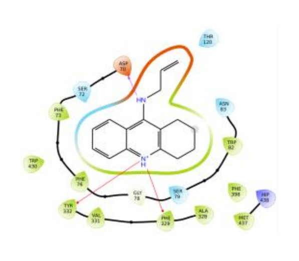	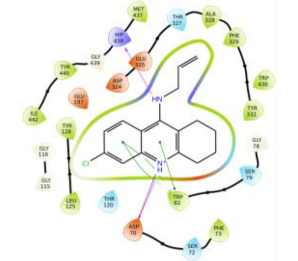	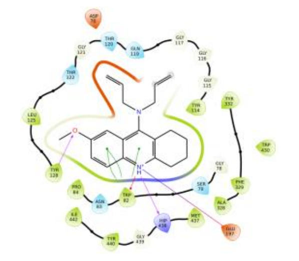
**5**-*h*BChE	**6**-*h*BChE	**7**-*h*BChE	**8**-*h*BChE

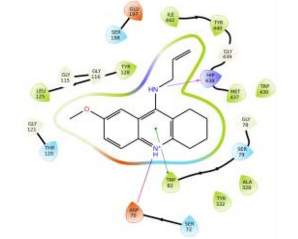	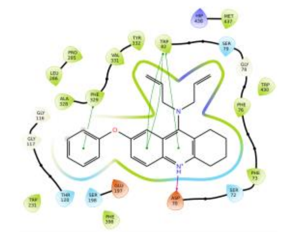	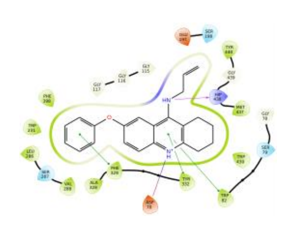	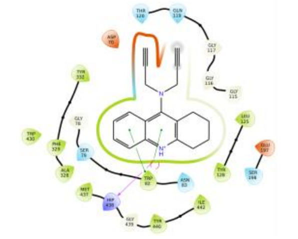
**9**-*h*BChE	**10**-*h*BChE	**11**-*h*BChE	**12**-*h*BChE
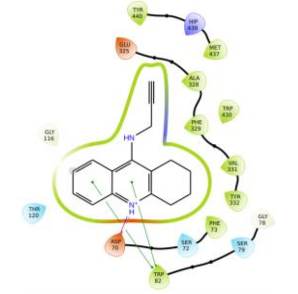	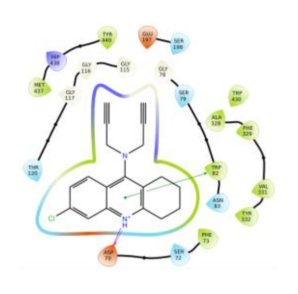	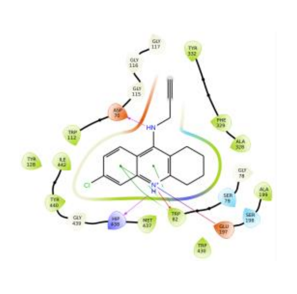	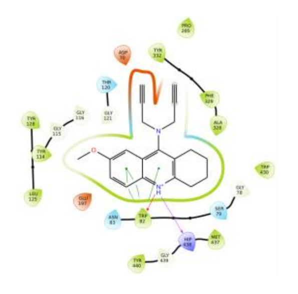
**13**-*h*BChE	**14**-*h*AChE	**15**-*h*BChE	**20**-*h*BChE
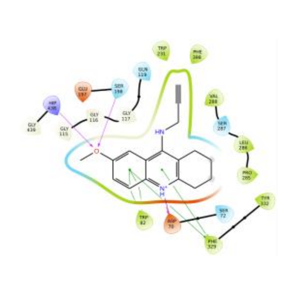	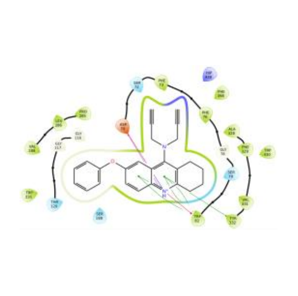	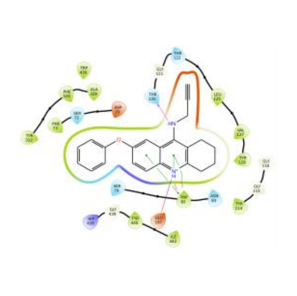	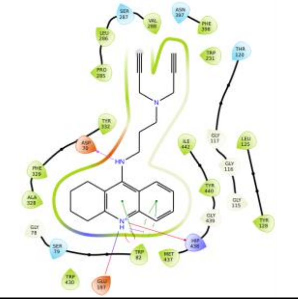
**21**-*h*BChE	**22**-*h*BChE	**23**-*h*BChE	**24**-*h*BChE
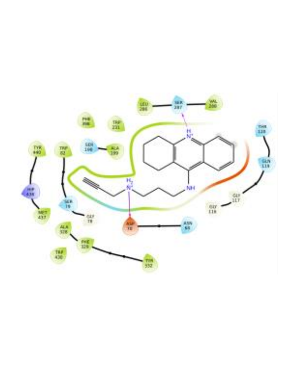	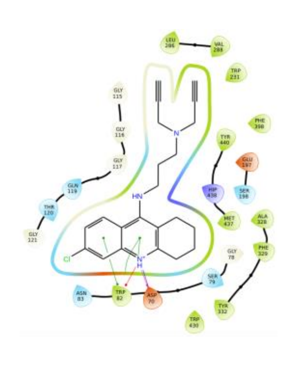	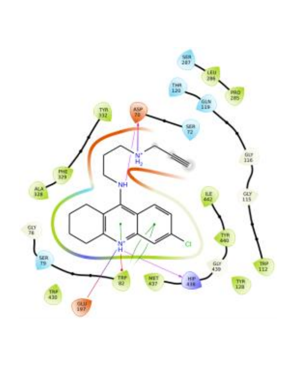	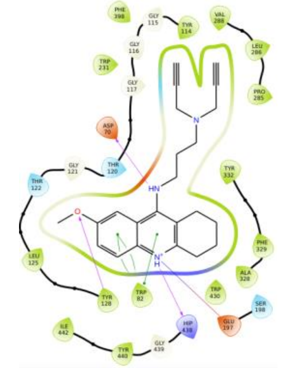
**25**-*h*BChE	**26**-*h*BChE	**27**-*h*BChE	**28**-*h*BChE
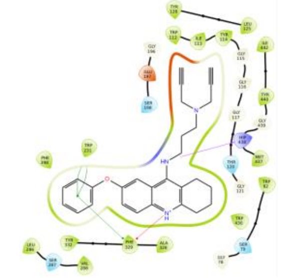	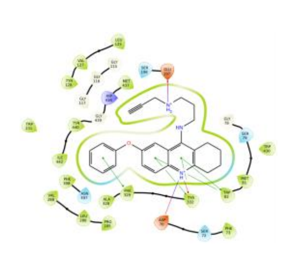	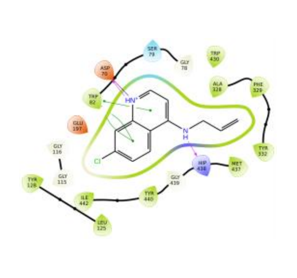	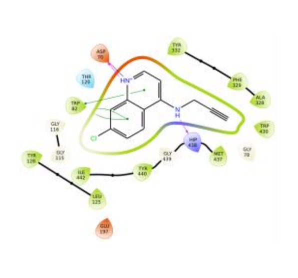
**THA**-*h*BChE	**6-Cl-THA**-*h*BChE	**7-MEOTA**-*h*BChE	**7-PhOTHA**-*h*BChE

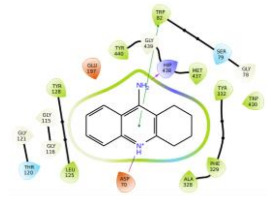	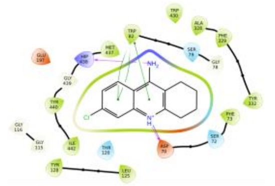	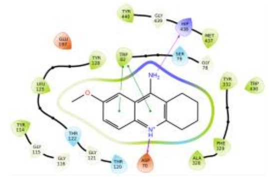	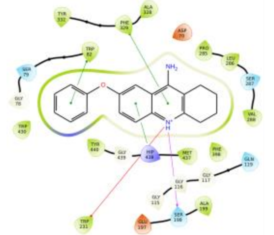
**clorgyline**-*h*BChE	**pargyline**-*h*BChE		
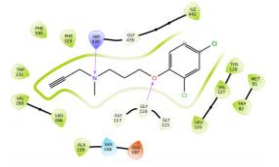	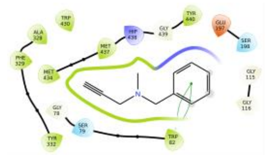		

Table S6. Predicted top scoring binding modes of the studied compounds in *h*MAO-A (PDB ID: 2Z5X) by IFD with OPLS_2005 force field in Schrodinger 2021-4.

**1**-*h*MAO-A	**2**-*h*MAO-A	**3**-*h*MAO-A	**4**-*h*MAO-A
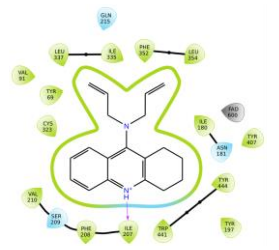	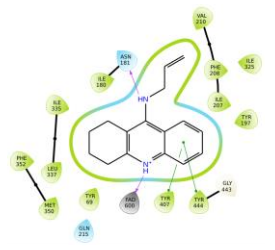	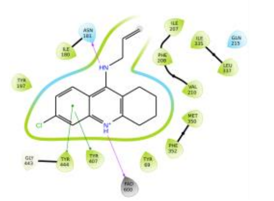	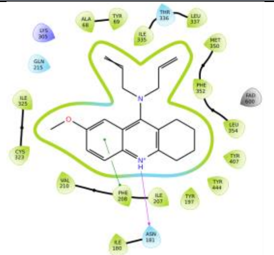
**5**-*h*MAO-A	**6**-*h*MAO-A	**7**-*h*MAO-A	**8**-*h*MAO-A
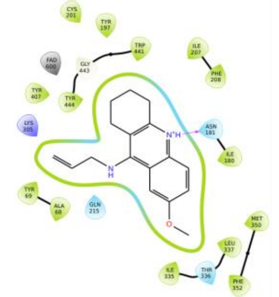	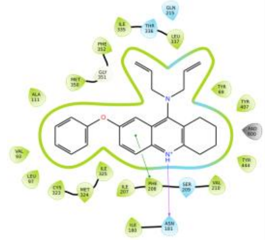	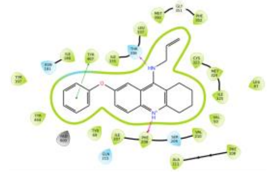	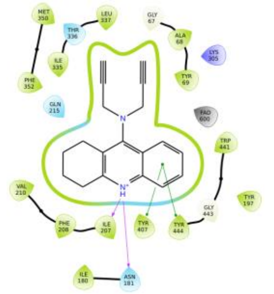
**9**-*h*MAO-A	**10**-*h*MAO-A	**11**-*h*MAO-A	**12**-*h*MAO-A
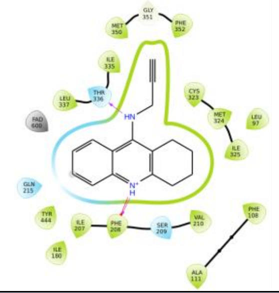	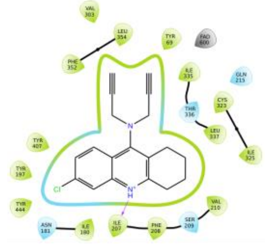	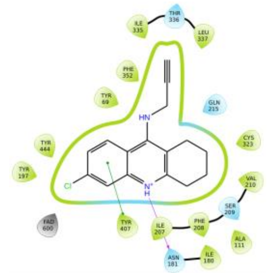	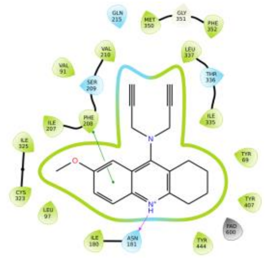
**13**-*h*MAO-A	**14**-*h*MAO-A	**15**-*h*MAO-A	**20**-*h*MAO-A

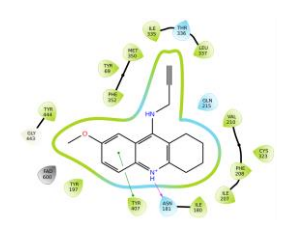	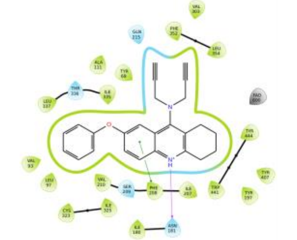	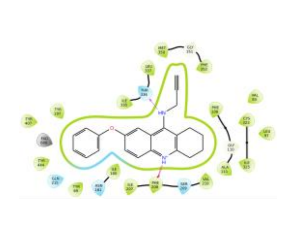	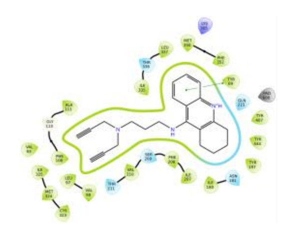
**21**-*h*MAO-A	**22**-*h*MAO-A	**23**-*h*MAO-A	**24**-*h*MAO-A
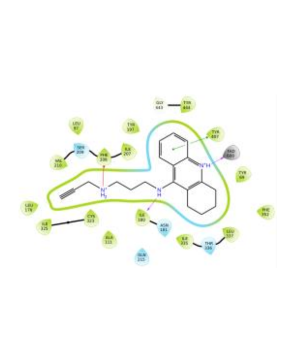	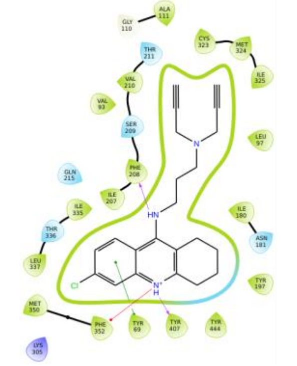	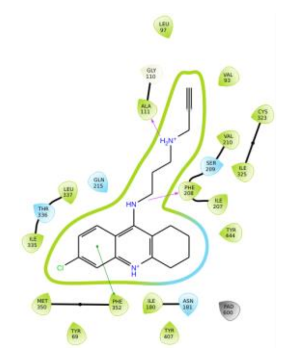	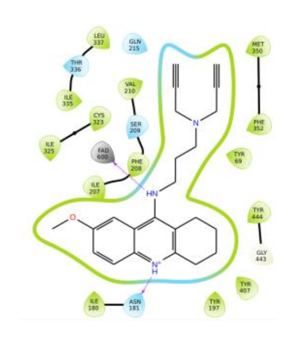
**25**-*h*MAO-A	**26**-*h*MAO-A	**27**-*h*MAO-A	**28**-*h*MAO-A
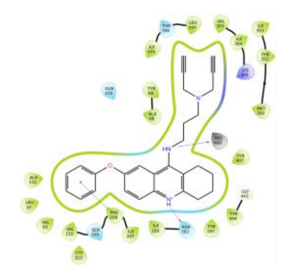	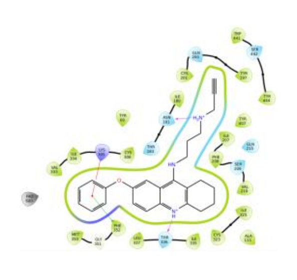	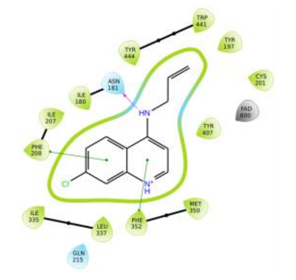	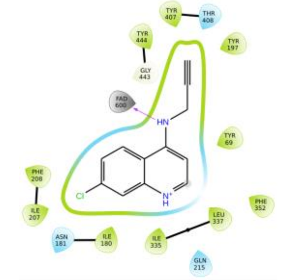
**THA**-*h*MAO-A	**6-Cl-THA**-*h*MAO-A	**7-MEOTA**-*h*MAO-A	**7-PhOTHA**-*h*MAO-A
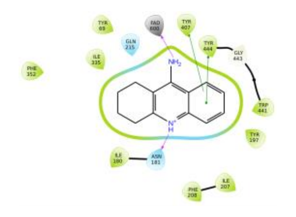	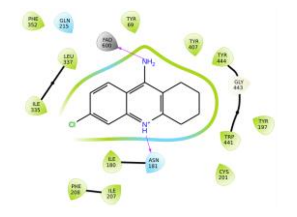	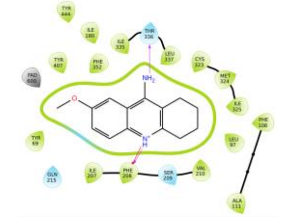	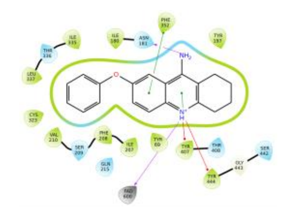
**clorgyline**-*h*MAO-A	**pargyline**-*h*MAO-A		
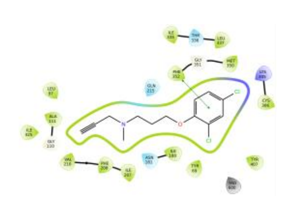	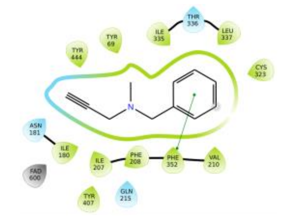		

Table S7. Predicted top scoring binding modes of the studied compounds in *h*MAO-B (PDB ID: 3PO7) by IFD with OPLS_2005 force field in Schrodinger 2021-4.

**1**-*h*MAO-B	**2**-*h*MAO-B	**3**-*h*MAO-B	**4**-*h*MAO-B

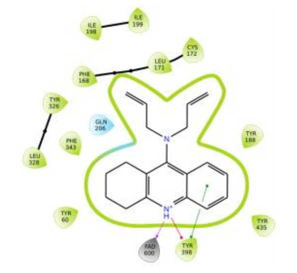

5-*h*MAO-B

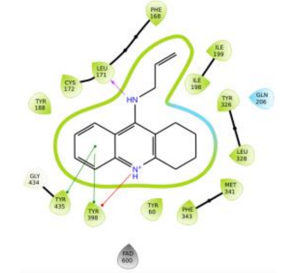

6-*h*MAO-B

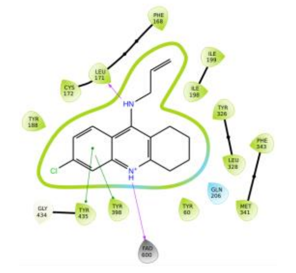

7-*h*MAO-B

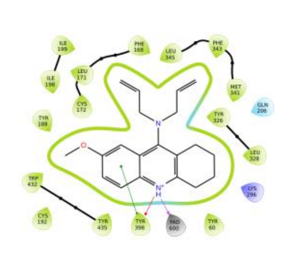

8-*h*MAO-B

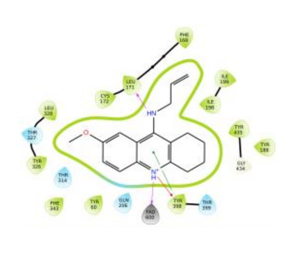

9-*h*MAO-B

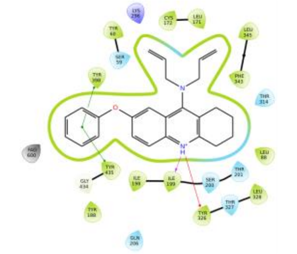

10-*h*MAO-B

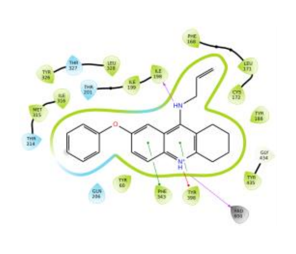

11-*h*MAO-B

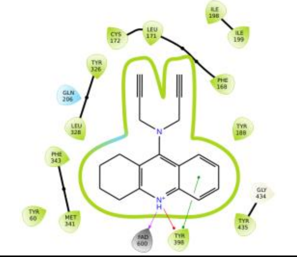

12-*h*MAO-B

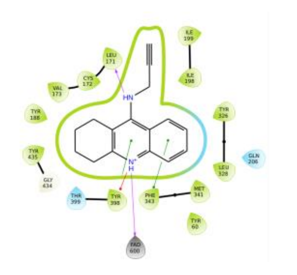

13-*h*MAO-B

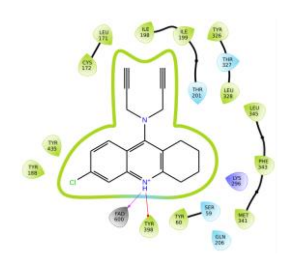

14-*h*MAO-B

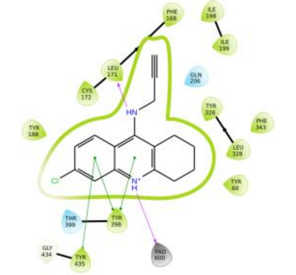

15-*h*MAO-B

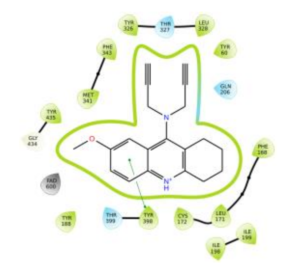

20-*h*MAO-B

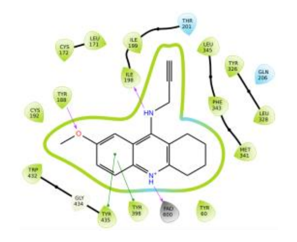

21-*h*MAO-B

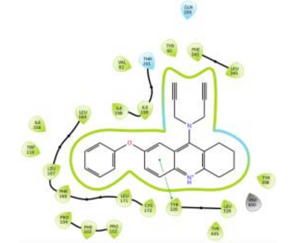

22-*h*MAO-B

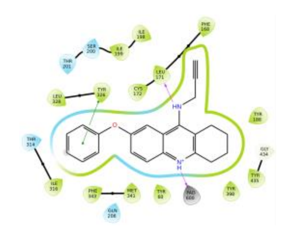

23-*h*MAO-B

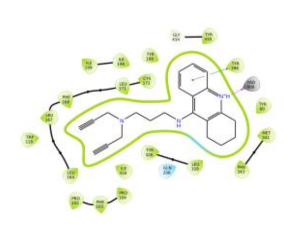

24-*h*MAO-B

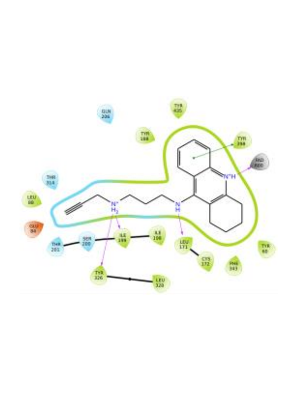

25-*h*MAO-B

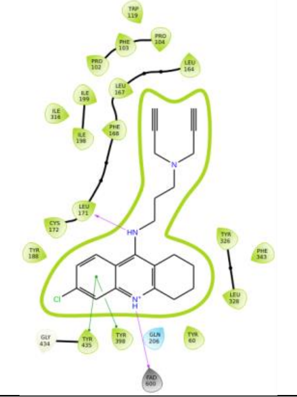

26-*h*MAO-B

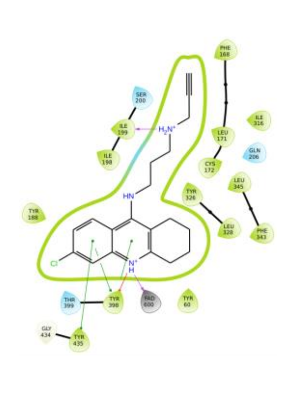

27-*h*MAO-B

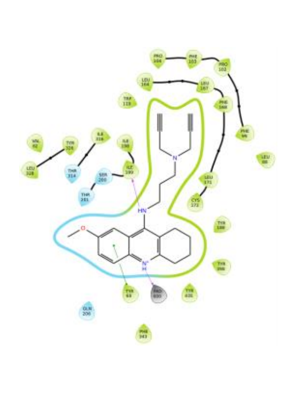

28-*h*MAO-B

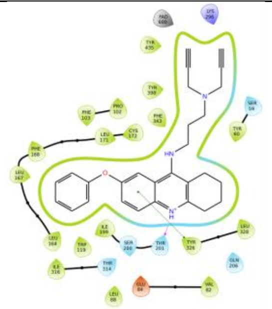	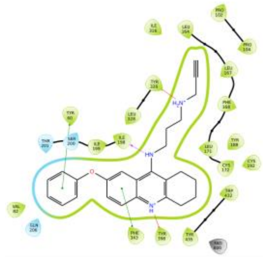	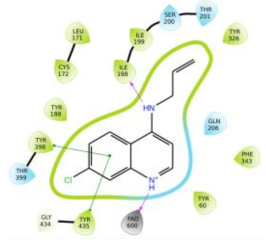	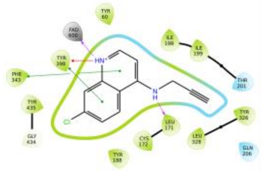
**THA**-*h*MAO-B	**6-Cl-THA**-*h*MAO-B	**7-MEOTA**-*h*MAO-B	**7-PhOTHA**-*h*MAO-B
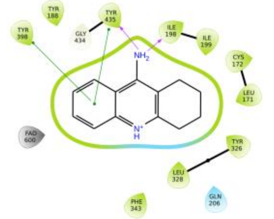	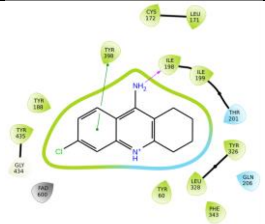	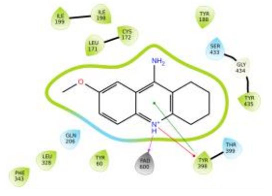	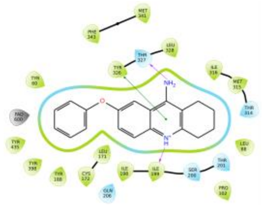
**clorgyline**-*h*MAO-B	**pargyline**-*h*MAO-B		
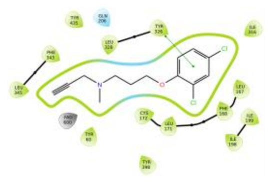	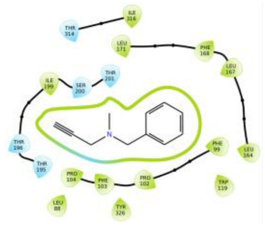		
